# Different drivers, common mechanism; the distribution of a reef fish is restricted by local-scale oxygen and temperature constraints on aerobic metabolism

**DOI:** 10.1093/conphys/coaa090

**Published:** 2020-10-26

**Authors:** Murray I Duncan, Nicola C James, Warren M Potts, Amanda E Bates

**Affiliations:** 1 Department of Ichthyology and Fisheries Science, Rhodes University, Prince Alfred street, Makhanda, 6140, South Africa; 2 South African Institute for Aquatic Biodiversity, 11 Somerset street, Makhanda, 6139, South Africa; 3 Department of Ocean Sciences, Memorial University of Newfoundland, 0 Marine Lab Rd, St. John’s, NL, A1C 5S7, Canada

**Keywords:** Climate change, distribution, fisheries, metabolic index, oxygen, temperature

## Abstract

The distributions of ectothermic marine organisms are limited to temperature ranges and oxygen conditions that support aerobic respiration, quantified within the metabolic index (ϕ) as the ratio of oxygen supply to metabolic oxygen demand. However, the utility of ϕ at local scales and across heterogenous environments is unknown; yet, these scales are often where actionable management decisions are made. Here, we test if ϕ can delimit the entire distribution of marine organisms when calibrated across an appropriate temperature range and at local scales (~10 km) using the endemic reef fish, *Chrysoblephus laticeps*, which is found in the highly heterogenous temperature and oxygen environment along the South African coastal zone, as a model species. In laboratory experiments, we find a bidirectional (at 12°C) hypoxia tolerance response across the temperature range tested (8 to 24°C), permitting a piecewise calibration of ϕ. We then project this calibrated ϕ model through temperature and oxygen data from a high spatial resolution (11 to 13 km) ocean model for the periods 2005 to 2009 and 2095 to 2099 to quantify various magnitudes of ϕ across space and time paired with complementary *C. laticeps* occurrence points. Using random forest species distribution models, we quantify a critical ϕ value of 2.78 below which *C. laticeps* cannot persist and predict current and future distributions of *C. laticeps* in line with already observed distribution shifts of other South African marine species. Overall, we find that *C. laticeps*’ distribution is limited by increasing temperatures towards its warm edge but by low oxygen availability towards its cool edge, which is captured within ϕ at fine scales and across heterogenous oxygen and temperature combinations. Our results support the application of ϕ for generating local- and regional-scale predictions of climate change effects on organisms that can inform local conservation management decisions.

## Teaser

The metabolic index (ϕ) captures how temperature and oxygen interact to limit marine organism distributions at local scales. These findings indicate that ϕ has utility as a conservation tool for local management.

## Introduction

Climate change is driving the redistribution of species across the globe (Poloczanska *et al.*, 2013; Pecl *et al.*, 2017). Marine species are particularly responsive to climate-driven distribution shifts because they are exposed to environmental conditions closer to their limits and have fewer colonization barriers than their terrestrial counterparts (Sunday *et al.*, 2012; Pinsky *et al.*, 2019). The general warming trend of the ocean has, on a broad scale, already driven the distribution of fishes to higher latitudes (Perry *et al.*, 2005) or deeper depths (Dulvy *et al.*, 2008), but this directional pattern is far from ubiquitous (Pinsky *et al.*, 2020). Predicting the direction and magnitude of climate-driven distribution shifts of marine organisms is important to guide management and conservation strategies (Brander, 2007; Link *et al.*, 2011) but is complicated by variable, species-specific distribution responses to prevailing climatic conditions (Pinsky *et al.*, 2020). Identifying the fundamental physiological mechanisms governing how organisms respond to environmental variability may overcome these complications and improve predictive accuracy (Evans *et al.*, 2015).

Ectothermic organism performance is regulated by the environment through its influence on internal physiological rates and processes (Bozinovic and Pörtner, 2015; Horodysky *et al.*, 2015). All physiological and biochemical processes where energy is consumed or stored are expressed as an organism’s metabolism (Nelson, 2016). These energy conversions are fuelled by ATP (adenosine triphosphate) which is generated up to 30 times more efficiently through aerobic versus anaerobic metabolic pathways (Richards, 2009; Clarke, 2019). Any constraints to an organism’s aerobic metabolism thus correspond to reductions in overall energy available to provision processes related to performance. When localized environmental conditions change, organisms can seek areas where rates of aerobic metabolism can be maintained, ultimately resulting in a net distribution shift (Walther *et al.*, 2002; Parmesan, 2006; Cheung *et al.*, 2009). Determining the environmental conditions that constrain aerobic metabolism is therefore an important physiological consideration that can improve interpretability and forecasting accuracy of distribution shifts (Kearney and Porter, 2009; Teal *et al.*, 2018; Rodríguez *et al.*, 2019).

Temperature and oxygen availability are the primary controlling and limiting factors on the aerobic metabolism of ectotherms (Claireaux and Chabot, 2016) and are also the two environmental variables most pervasively influenced by anthropogenic climate change (Gruber, 2011). Temperature regulates the rate of aerobic metabolism as more energy is required for physiological processes driven faster by increasing kinetic energy at high temperatures (Clarke and Fraser, 2004). Oxygen availability poses an upper limit on aerobic metabolism, as the rate at which an organism can consume the required oxygen to fuel physiological processes is set by rates of diffusion via a pressure gradient from the environment across gill epithelium (Ern, 2019). The temperature effect on aquatic ectotherm aerobic metabolism is often depicted as absolute or factorial aerobic scope [the difference or ratio between the maximum (MMR) and standard metabolic rates (SMR) respectively] and is thought to represent the excess metabolic energy above that required for maintenance at a given temperature (Fry, 1971; Farrell, 2016). The oxygen- and capacity-limited thermal tolerance (OCLTT) hypothesis posits that the often-bell-shaped aerobic scope–temperature relationships are driven by differences in oxygen supply and oxygen demand and define the thermal envelope within which an organism can persist (Portner and Farrell, 2008). There is, however, mounting evidence against the universality of the OCLTT hypothesis, including mismatches between thermal optima for aerobic scope and various important ecological and physiological processes (Gräns *et al.*, 2014; Norin *et al.*, 2014; Verberk *et al.*, 2016). Whilst the OCLTT concept suggests that thermal limits are set by failures in the oxygen delivery system, it does not explicitly incorporate how this system is influenced by ambient oxygen availability, which together with temperature are the primary variables governing metabolism. A quantitative model that can evaluate how both these environmental variables interact to drive an aquatic organisms’ aerobic metabolism has only relatively recently been developed, in the form of the metabolic index (ϕ) (Deutsch *et al.*, 2015).

The metabolic index (ϕ) represents the ratio of oxygen supply to resting oxygen demand, incorporating the effect of both temperature and oxygen availability. Oxygen demand is quantified as the critical oxygen partial pressure (*p*O_2crit_) which represents the minimum level of oxygen availability to sustain a standard metabolic rate (Ultsch and Regan, 2019) and oxygen supply is taken as the prevailing oxygen partial pressure (*p*O_2_). The critical *p*O_2_ for maximum metabolic rates of aquatic organisms has evolved to match prevailing *p*O_2_ and thus at normoxia ϕ is akin to factorial aerobic scope (MMR/SMR) (Seibel and Deutsch, 2020). Unlike factorial aerobic scope, ϕ quantitatively incorporates oxygen availability together with temperature effects on aerobic metabolism and offers a powerful framework to explore how variability in these two environmental variables can control the distribution of viable habitat for a species (Somero *et al.*, 2016). Indeed, Deutsch *et al*. (2015) showed that a mean ϕ threshold (ϕ_crit_) ranging between 2 and 5 limits the equatorward distribution of four marine species from diverse habitats. Penn *et al*. (2018) argue that historical temperature and oxygen levels at the end of the Permian Period exceeded ϕ_crit_, accounting for the spatial variation of mass marine extinctions at the time. These studies explored spatial trends in ϕ across broad north to south extents and from coarse-resolution ocean models, with the patterns observed largely attributed to increasing temperature driving metabolic demand above supply.

At local scales, organisms respond to abrupt environmental heterogeneity rather than broader climate signals, which occur on scales of metres to kilometres, particularly around the coastal zone (Bates *et al.*, 2018). For example, localized upwelling can pull deeper oxygen-deprived water towards the surface resulting in anomalously low coastal oxygen partial pressures and sea temperatures compared to surrounding coastal waters (Lutjeharms *et al.*, 2000; Grantham *et al.*, 2004). Here, we use the considerable spatial variability in temperature and oxygen availability along South Africa’s coastal zone (Roberts, 2005) as the model system to explore if ϕ can explain the contemporary distribution patterns of an endemic marine fish (*Chrysoblephus laticeps*) and predict future distribution responses. We hypothesize that ϕ will be limited towards (1) the warm-temperate edge of *C. laticep’s* distribution through a temperature-induced increase in oxygen demand and (2) the cool-temperate edge through the low oxygen availability in this region.

## Methods

### Study area and species profile

The South African coastal zone is characterized by a significant local-scale spatial variability in temperature and oxygen availability signals making it a model system to explore how these two important environmental variables may interact to limit species distributions. The coastal zone is subdivided into three main bioregions (cool-temperate, warm-temperate and subtropical) with these characterized by spatial and temporal heterogeneous ocean temperatures and oxygen availability (Potts *et al.*, 2015). The east coast is subtropical, with coastal temperatures driven by the warm Agulhas current, which has intensified and warmed since the 1980s (Rouault *et al.*, 2009). Oxygen concentration is high (>4 ml.l^−1^) relative to temperatures throughout most parts of the east coast (Pretorius *et al.*, 2016). By contrast, the south coast is warm-temperate and is characterized by temperature variability patterns driven by intermittent upwelling (Goschen and Schumann, 2011). Although there is a negative trend in mean annual sea surface temperature along areas of the south coast associated with increases in upwelling (Rouault *et al.*, 2010), some localized areas of warming are also reported (Lima and Wethey, 2012). Oxygen availability is relatively high throughout the south coast (>4 ml.l^−1^), but there are localized areas (towards the west of the south coast) where oxygen levels can get to critically low (0–2 ml.l^−1^) levels (Roberts, 2005). The south-west coast is cool-temperate, with environmental patterns dominated by the more permanent Benguela upwelling cell that is situated north of Cape Town. Sea temperatures are cooler than the south coast, and oxygen concentrations are low (0–2 ml.l^−1^) (Roberts, 2005; Jarre *et al.*, 2015). Here, the Benguela upwelling has strengthened since the 1970s resulting in a negative mean annual sea surface temperature trend (Santos *et al.*, 2012).

We used the roman seabream (*Chrysoblephus laticeps*) as a model species for this study. Typical of most species in the sparid genus, *C. laticeps* is considered slow growing, has a maximum age of 19 years (Götz *et al.*, 2008), attains 50% maturity as a female between ages 2.5 and 4.27 (Buxton 1987, 1990, Götz *et al*. 2008), and undergoes a protogynous sex change (Buxton, 1990) between ages 8 and 10.25 (Götz *et al.*, 2008). Importantly, this species is highly resident, with the probability of being recaptured within the Tsitsikamma National Park Marine Protected Area estimated at 0.94 (Kerwath *et al.*, 2007a) and a home range size estimated using acoustic telemetry of between 1 and 3 km^2^ (Kerwath *et al.*, 2007b). Because *Chrysoblephus laticeps* forms an important component of the commercial fisheries in South Africa, a high-resolution spatial data of where the species is caught available (Kerwath *et al.*, 2013).

### Calibrating the metabolic index for *Chrysoblephus laticeps*

The metabolic index (ϕ) (Equation 1) represents the ratio of oxygen supply to oxygen demand where supply is the prevailing oxygen partial pressure (*p*O_2_) and demand is the minimum *p*O_2_ required to sustain a standard metabolic rate (*p*O_2crit_) taking into account temperature and mass (Deutsch *et al.*, 2015).

(1) \begin{equation*}\phi ={A}_{\mathrm{o}}{B}^n\frac{P{O}_2}{\exp \Big(\frac{-{E}_{\mathrm{o}}}{k_BT}\Big)}\end{equation*}

The parameters of ϕ (*A*_o_ and −*E*_o_) are derived from the slope and intercept of the linear relationship between critical oxygen partial pressure (*p*O_2crit_) which has been mass standardized [divided by mass (*B*) and its scaling exponent (*n*)] and the inverse of *k_B_T* [the product of temperature (T) in kelvin and the Boltzmann constant (*k_B_*)]. These parameters are species-specific and thus need to be derived from laboratory experiments that quantify a species’ *p*O_2crit_ across a temperature range.

Fifty *C. laticeps* specimens were caught from either the Tsitsikamma Marine Protected Area (*n* = 25, date = 23 September 2016) or offshore of the Noordhoek Ski-boat Club outside of Port Elizabeth (*n* = 25, date = 20 September 2016) and brought back alive to the Aquatic Ecophysiology Research Platform laboratories of the South African Institute for Aquatic Biodiversity. Fish were left to adjust for ~6 weeks to holding tank conditions kept at the average bottom sea temperature of sampling locations (16°C) before standard and maximum metabolic rates were quantified. These metabolic rate measurements formed part of a complimentary study quantifying the metabolic response of *C. laticeps* to abrupt temperature swings which are a common occurrence through its distribution (see Duncan *et al*. 2019 for detailed methods). The protocol involved placing fasted individuals inside respirometers at holding temperatures (16°C), allowing ~12 h to adjust to respirometer conditions and then subjecting them to an acute (1°C per hour) temperature change to either 8, 12, 16 (no change), 20 or 24°C test temperatures. At these test temperatures, intermittent flow respirometry (15-min flush, 5-min measure) was run for ~20 h whereafter individuals were chased to exhaustion, exposed to air and placed back inside the respirometer to elicit maximum metabolic rate. The standard metabolic rate was determined as the quantile that assigned the bottom 20% of all metabolic rate measurements prior to the chase protocol (Chabot *et al.*, 2016). Following the elicitation of maximum metabolic rate each individual was left in its respirometer and given time to recover (~4 to 5 h) to metabolic rates approximating its standard metabolic rate. At this point, the protocol to determine the critical oxygen saturation (O_2crit_) was started. Respirometer flush pumps were turned off, and oxygen was depleted within a respirometer by the organism until distinct signs of stress or impaired performance were observed. Metabolic rates were measured in 5-min intervals during this progressive hypoxia and combined with all earlier metabolic rate measurements to quantify the critical oxygen level.

O_2crit_ was defined as the oxygen saturation level where standard metabolic rates could no longer be maintained and below which metabolic rates decreased in proportion with prevailing oxygen saturation levels. To quantify O_2crit_, we used the ‘calcO2crit’ function, developed by Claireaux & Chabot (2016), where O_2crit_ is taken as the point of intersection between standard metabolic rate and the linear declines in metabolic rates below the standard metabolic rate in hypoxic water (Figure S1.1 in the Supporting Information). First, all metabolic rate measurements (mg O_2_.min^−1^.kg^−1^) from Duncan *et al*. (2019) were combined with metabolic rate measurements during progressive hypoxia (this study) and paired with the average oxygen saturation of respirometer water during each measurement period. The ‘calcO2_crit_’ function was run for each individual dataset with the maximum number of points for the regression below the standard metabolic rate set to 15 to limit the potential for including too many points above ‘true O2_crit_’ and overestimating. Subsequent O2_crit_ plots were visually inspected, and O2_crit_ was considered overestimated if a large number of points were included in the regression below SMR, but metabolic rates were not decreasing in proportion to oxygen saturation levels. This can happen if metabolic rate measurements greater than standard metabolic rate are lacking at low oxygen levels and, in such cases, the maximum number of points was limited to four (one more than the minimum required for a regression). All O2_crit_ thresholds (% saturation) were converted to corresponding critical oxygen partial pressures (*p*O_2crit_, kPa).

To standardize mass effects on *p*O_2crit_, we estimated the mass scaling exponent (*n*) for *C. laticeps* by first temperature standardizing data (done by dividing with the Arrhenius function) and finding the best fit mass scaling exponent from the mass–*p*O_2crit_ power-law relationship (Deutsch *et al.*, 2015). To test for differences in *p*O_2crit_ between the Tsitsikamma (TNP) and Port Elizabeth (PE) sampling areas, the linear relationship between *p*O_2crit_ and temperature was modelled including area as an additive effect and its interaction with temperature, but only for temperatures between 12 and 24°C to ensure the residuals of the linear model were normally distributed. We also followed the same approach to determine if sex had a significant effect on *p*O_2crit_. The sex and stage of each study organism were determined by inspecting the gonads of euthanized individuals following the O_2crit_ trial. Without microscopic gonad sections, it was difficult to determine the sexual stage (male, female or intersex) with 100% accuracy. Sex was therefore classified as either female (F), male (M), predominately male but possibly intersex stage (M/I) or predominately female but possibly intersex stage (F/I), and included as an additive and interactive effect with temperature in a linear model with *p*O_2crit_ as the dependent variable_._ If no mass and sex stage effects were found, all data were pooled to estimate the parameters of ϕ.

### Environmental and occurrence data

Spatial environmental data were obtained from a high-resolution (0.25° horizontal resolution) coupled ocean-biogeochemistry model (Yool *et al.*, 2015; Popova *et al.*, 2016) for the periods between 2005 and 2009 (contemporary) and projections towards 2095–2099 (future). The model framework is built on the physical Nucleus for European Modelling of the Ocean (NEMO) model (Madec, 2008) coupled with a Model of Ecosystem Dynamics, Nutrient Utilisation, Sequestration and Acidification (MEDUSA 2.0) (Yool *et al.*, 2013). The forward projection is based on the IPCC representative concentration pathway 8.5, which is the worst case, business-as-usual scenario of future greenhouse gas concentrations (IPCC, 2014).

Mean monthly contemporary and future oxygen concentration (mmol.m^−3^), temperature (°C), salinity (psu) and depth data were extracted from the layer of cells closest to the sea floor because *C. laticeps* is a benthic reef-associated species. Data were extracted for depths between 0 and 100 m below sea level and from around the southern African coast (26.5–37.5 °S and 13.5–37.5 °E). Oxygen concentrations were converted to partial pressure (kPa) based on grid cell temperature, oxygen concentration, salinity and pressure at depth using the ‘o2_unit_conv’ function in the ‘presens’ package (Birk, 2016). Pressure at depth was independently determined based on grid cell depth and latitude using the ‘swPressure’ function in the ‘oce’ package (Kelly and Richards, 2018). The mean of surrounding cells was used to estimate values for those cells with no data near the coast and the resolution of environmental data was increased to 0.125° horizontal resolution (13.31–11.27 km) by locally resampling using bilinear interpolation with the ‘raster’ package (Hijmans, 2016). Monthly ϕ grid cells were generated by projecting the calibrated ϕ equation across the spatial and temporal extent of temperature and oxygen partial pressure data from the ocean model. Monthly mean, minimum and maximum ϕ layers were developed for contemporary (2005–2009) and future (2095–2099) model predictions. Gridded bathymetry data were obtained from the General Bathymetric Chart of the Oceans (GEBCO) website at a 30 arc-second resolution and resampled to a 0.125° horizontal resolution (13.31–11.27 km) by taking the mean depth of every cell within the 0.125° grid and clipped between 0 and 100 m below sea level.

Occurrence points for *C. laticeps* were obtained from the National Marine Linefish System (NMLS) for the period between 2000 and 2010, made available by The Department of Environment, Forestry and Fisheries [DEFF, formally the Department of Agriculture Fisheries and Forestry (DAFF)]. The NMLS contains mandatory species level catch and effort data from the boat-based commercial fisheries operating in South African waters since 1985 and is one of the largest geo-referenced marine datasets in the world (DAFF, 2016). The boat-based fishery operates throughout South Africa’s coastal zone, and we are confident that the occurrence points from the NMLS accurately represent where *C. laticeps* occurs.

### Distribution modelling

We used the random forest algorithm (Breiman, 2001) to model the current distribution, quantify critical ϕ levels (ϕ_crit_) and predict future distributions of *C. laticeps* up to 2099 using the ‘randomForest’ package (Liaw and Weiner, 2002). Random forests are an extension of traditional classification trees, where many trees are grown using bootstrapped samples of data, and a random selection of predictor variables at each tree node is used for classification (Cutler *et al.*, 2007). We used random forests because they are not especially sensitive to predictor collinearly or the distribution of data and are commonly used and considered accurate for species distribution modelling (Cutler *et al.*, 2007; Mi *et al.*, 2017).

We followed the guidelines of Barbet-Massin *et al*. (2012) for applying random forests to model the distribution of *C. laticeps*. A dataset consisting of the known occurrence points of *C. laticeps* was combined with an equal number of pseudo-absence data points. Pseudo-absence data points were generated by randomly selecting cells throughout the extent of the predictor variables but excluding cells adjacent to known occurrence points (Barbet-Massin *et al.*, 2012). This presence–pseudo-absence dataset was then randomly subdivided into a model training (80%) and a model testing (20%) dataset. The predictive performance of each model was assessed based on the accuracy of correctly classifying the test dataset and variable importance of the predictors ranked according to the mean decrease in accuracy based on random permutations of each predictor variable.

To assess what value of ϕ may limit the distribution of *C. laticeps* (ϕ_crit_), 10 full random forest models were trained and assessed with maximum, minimum and mean monthly ϕ, and depth as predictor variables, for the contemporary period. Each of the 10 models was trained with a unique generation of pseudo-absence data and subsequent random training/testing data split. Ten more reduced random forest models were then re-trained and tested using only the top two predictor variables from the full models. The predictive accuracy (measured as a proportion of correctly identified test data cells) of the full versus the reduced models were compared and the simplest combination of predictor variables with the highest accuracy subsequently used to project current and future distributions of *C. laticeps*. Current and future distributions of *C. laticeps* were estimated by combining all 10 model projections, trained on unique combinations of presence–pseudo-absence data (described above), with most confidence given to areas where all 10 models agreed. To quantify the critical threshold of ϕ (ϕ_crit_), the feature contribution (the contribution of a data point to the final model prediction) of each training data point was first extracted from the random forest models using the ‘forestFloor’ package (Welling *et al.*, 2016) for all 10 current model runs and combined. A feature can contribute either positively (towards an occurrence prediction) or negatively (towards an absence prediction) to the random forest model prediction. In order to fit a logistic regression through the ϕ feature contribution data, they were converted to binary data by reclassifying negative feature class contributions to zero and positive contributions to one. ϕ_crit_ was estimated as the ϕ value where the logistic regression model = 0.5. Other predictor variable thresholds were estimated as the points where the majority of feature class contributions transitioned from positive to negative.

### Comparing ϕ as a predictor variable to other common metrics

To compare where and how ϕ may differ from distribution predictions over other commonly used predictor variables, we followed the distribution modelling steps outlined above but for distribution models including minimum, maximum and mean values of either absolute aerobic scope (AAS), factorial aerobic scope (FAS) or prevailing temperature as predictor variables. Aerobic scope environmental layers were generated by fitting quadratic polynomials through the relationship between mass standardized standard (SMR) or maximum metabolic rates (MMR) and temperature, from data corresponding to the same individuals in this study but measured in Duncan *et al*. (2019), and projecting these models through the temperature layers of the ocean model. Absolute aerobic scope was taken as MMR minus SMR for a given temperature grid cell and FAS taken as MMR divided by SMR. For each predictor variable (AAS, FAS or temperature), we ran 10 random forest models with minimum, mean and maximum values from the transformed ocean model as predictor variables and added model outputs together resulting in spatial distribution predictions ranging from 100 (all 10 models agree for the area to be suitable) to 0 (no models indicate area to be suitable).

**Figure 1:**
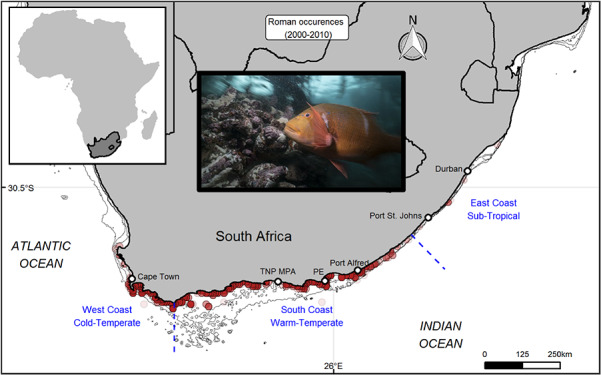
Map of study area. Major biogeographical zones (blue text) delineated by blue dashed lines, depth contours (50, 100 and 150 m below sea level, solid black lines) and land referenced localities referred to in text (white points) along the South African coastal zone. The image insert is the study species; *Chrysoblephus laticeps* (photo credit: Steven Benjamin www.animalocean.co.za) and red points represent known occurrences of *C. laticeps* obtained from the National Marine Linefish System for the period between 2000 and 2010.

## Results

### *p*O_2crit_ experiments

Of the 50 specimens obtained from the wild, we ran 39 (20 from PE and 19 from TNP) O_2crit_ trials across the temperature range tested (Table S1.1 in the Supporting Information). Specimen mass ranged from 0.32 to 1.55 kg and was evenly distributed across temperature treatments. O_2crit_ appeared to be overestimated in only 5 of the 39 trials and was adjusted to four regression points accordingly (Table S1.1 in the Supporting Information). Of the 39 specimens, we identified 10 as males, 8 as female, 16 as females but possibly intersex and 3 as males but possibly intersex, and 2 were not identified (Table S1.1 in the Supporting Information). Sex classifications were evenly distributed among temperature treatments (Figure S1.4 in the Supporting Information).

**Figure 2:**
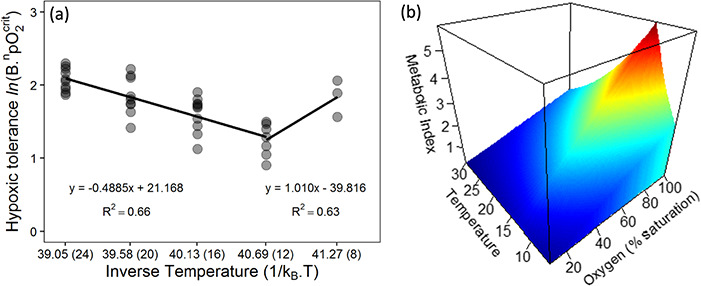
Calibrated metabolic index (ϕ) for *Chrysoblephus laticeps*. (**a**) Piecewise (12°C) relationship between the natural logarithm of mass standardised hypoxia tolerance [*ln*(B^n^.*p*O_2crit_)] and the inverse product of temperature (T in kelvin) and the Boltzmann constant (*k*_B_ in eV). Corresponding test temperatures in degrees celsius are indicated in brackets. (**b**) Graphical representation of ϕ for *C. laticeps* across a matrix of temperature and oxygen saturations.

**Figure 3:**
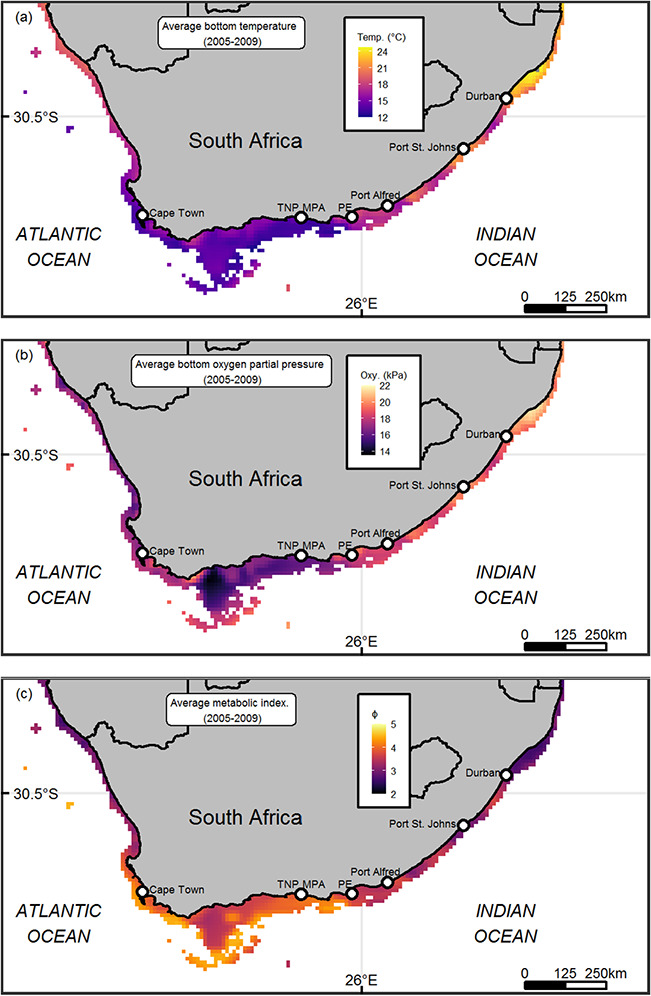
Spatial extent of environmental data and Metabolic Index (ϕ) for *Chrysoblephus laticeps*. (**a**) Average seafloor ocean temperature (°C) and (**b**) average seafloor oxygen partial pressure (kPa), obtained from the ocean model described in Section 2.3. (**c**) Average monthly metabolic index (ϕ) derived by projecting ocean model oxygen and temperature data through the laboratory calibrated ϕ equation described in Section 3.1.

### Calibration of the metabolic index

We identified a weak positive relationship between mass and *p*O_2crit_, with the mass scaling exponent estimated at 0.17 (Figure S1.2 in the Supporting Information). *p*O_2crit_ data revealed a positive linear relationship with temperature (12–24°C), which was not significantly different between sampling areas and their interaction with temperature (Tables S1.3, *P*-value > 0.05, Figure S1.3 in the Supporting Information) or sexual stage and its interaction with temperature (Tables S1.3, *P*-value > 0.05, Figure S1.4 in the Supporting Information). All data were subsequently pooled to determine the parameters of ϕ for *C. laticeps*. The differing slopes of the *p*O_2crit_–temperature relationship (8–12 and 12–24°C) permitted fitting a piecewise relationship to estimate ϕ parameters on either sides of 12°C [Fig. 2a, 12°C = 40.69 (1/*k_B_*.T)]. The estimated ϕ model parameters *A*_o,_  *E*_o_, taken from the intercept and slope respectively, could thus be estimated for each linear relationship. At temperatures greater than 12°C, we estimated *A*_o_ to be 1.55951E+9 and *E*_o_ to be −0.4885, and below 12°C *A*_o_ was estimated to be 5.109275E−18 and *E*_o_ to be 1.01. A graphical representation of this calibrated piecewise ϕ for *C. laticeps* is presented across a matrix of temperatures (**°**C) and oxygen levels (% saturation), indicating how temperature or oxygen availability can limit ϕ (Fig. 2b).

### Environmental and occurrence data

A total of 14 934 occurrence points were obtained and reduced to 206 points once duplicates were removed from the 0.125° grid cell resolution. Occurrence points were distributed throughout the South African south coast, in accordance with the published distribution of *C. laticeps* (Fig. 1).

Mean contemporary (2005–2009) spatial bottom temperature (Fig. 3a) and oxygen partial pressure (kPa) (Fig. 3b) were variable throughout the extent of the modelling domain. Mean current bottom temperatures ranged from 11.9 to 27.7°C and were the coolest along South Africa’s south coast but increasing in the east and west towards higher latitudes (Fig. 3a). The lowest monthly temperature of 10.33°C occurred in a cell off the south west coast in the Benguela upwelling zone, and the highest monthly temperature of 31.2°C occurred in a cell towards the north eastern extent of the environmental data. Mean contemporary oxygen availability ranged from 13.6 to 21.5 kPa, with low oxygen zones occurring offshore of South Africa’s west and central south coasts (Fig. 3b). The lowest monthly oxygen availability was 6.4 kPa and occurred along the south-west coast in the Benguela upwelling zone. The spatial variability in temperature and oxygen resulted in a heterogeneous spatial distribution ϕ for *C. laticeps* throughout the modelling domain (Fig. 3c). The mean ϕ for the period between 2005 and 2009 ranged from 2.17 to 4.78 and was the greatest along the south coast as warm temperatures along the east coast and low oxygen availability along the west coast limited ϕ.

### Species distribution modelling with ϕ

**Figure 4:**
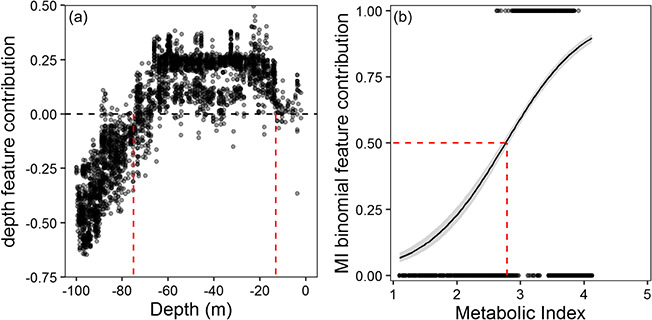
Modelled depth and metabolic index (ϕ) occurrence thresholds for *Chrysoblephus laticeps*. Model feature contributions (black dots) of depth cells (**a**) and minimum ϕ cells (**b**) from all 10 random forest reduced models. Depth thresholds for occurrence were between −75 and –13 m below sea level [red dashed lines in (a)] and the minimum ϕ threshold of 2.78 [red dashed lines in (b)] taken as the *x* value where minimum ϕ feature contribution = 0.5 from the logistic regression (solid black line) with 95% confidence intervals shaded grey.

The full random forest models based on all magnitudes (minimum, maximum and mean) of contemporary ϕ and depth had a mean predictive accuracy of 0.93 across all 10 runs. Depth and minimum ϕ were the two most important predictor variables in all full-model runs. The mean predictive accuracy of the reduced model with just depth and minimum ϕ as predictor variables was 0.92 and was statistically indistinguishable from the full models (student *T* test, *P*-value > 0.05, Table S1.4 in the Supporting Information). The reduced models were used to predict distribution changes and ϕ_crit_ for *C. laticeps*. Extracting contributions from parameters of the reduced models indicated that suitable depths for *C. laticeps* occur between around 13 and 75 m below sea level (Fig. 4a), and the threshold of minimum ϕ (ϕ_crit_) below which *C. laticeps* can no longer persist is 2.78 (Fig. 4b).

**Figure 5:**
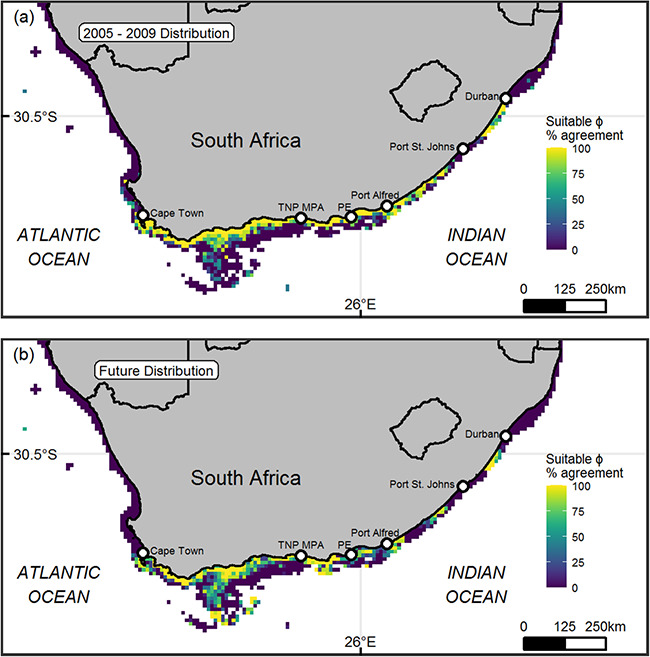
Modelled distributions of *Chrysoblephus laticeps*. (**a**) Modelled contemporary (2005–2009) and (**b**) future (2095–2099) distribution of *C. laticeps* based on an ensemble of 10 random forest projections for each time period. Areas where all 10 models predict suitable metabolic index (ϕ) levels are yellow.

The contemporary modelled distribution indicated a core range for *C. laticeps* from Cape Town in the west towards Port St. Johns in the east, with a break and a small patch of suitable habitat in the east (Fig. 5a). Projections indicate that this core distribution will persist up until 2100, but the western and eastern edges of the species distribution will contract slightly (Fig. 5b).

### Species distribution models with AAS, FAS and temperature

**Figure 6:**
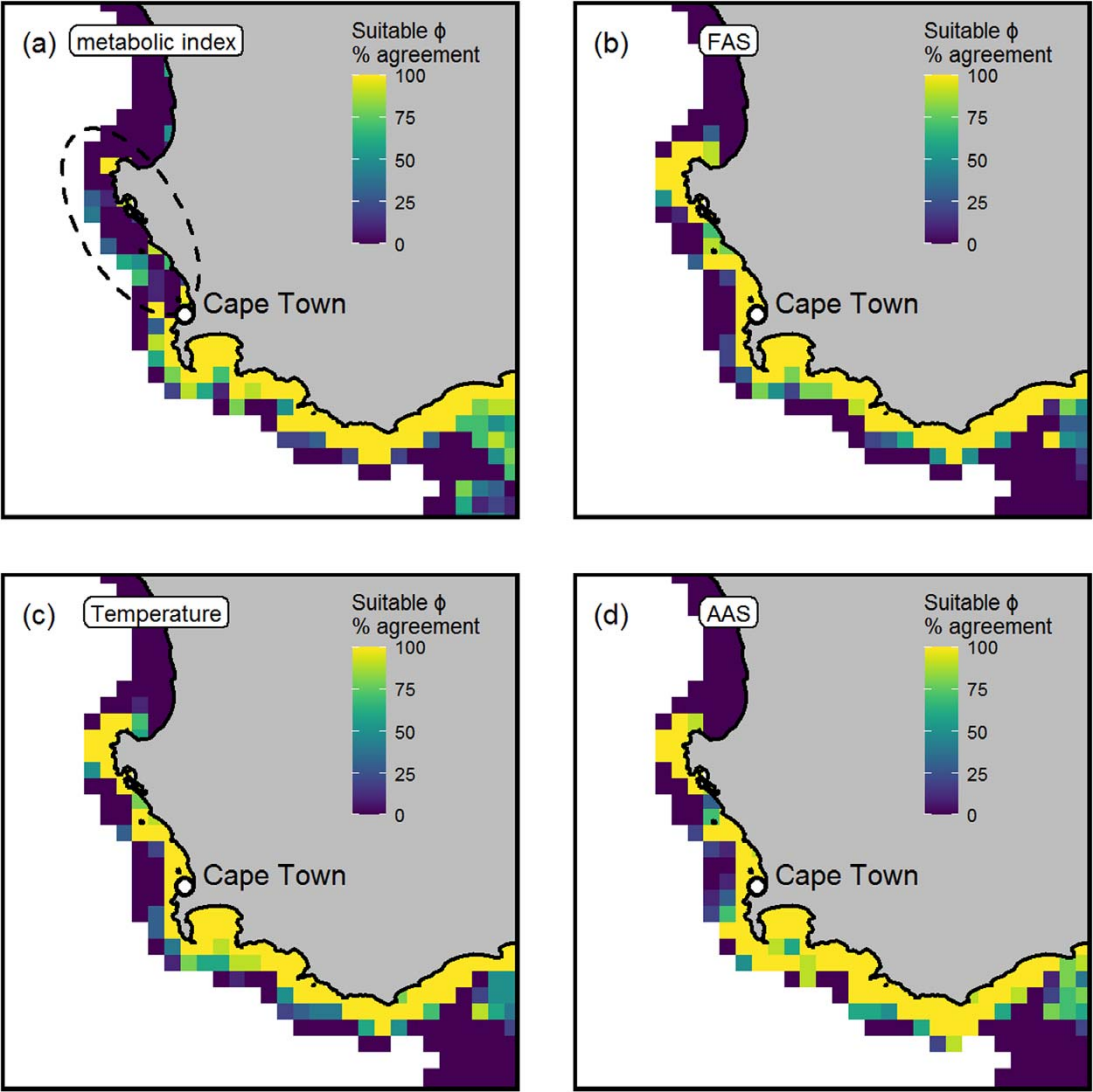
The metabolic index distribution model (**a**) better delimits the western edge of *Chrysoblephus laticeps’* distribution where catches are extremely rare (dashed ellipses) over other predictors. Modelled (2005–2009) distribution including metabolic index (a), factorial aerobic scope (FAS) (**b**), temperature (**c**) and absolute aerobic scope (AAS) (**d**) as predictor variables. Areas where all 10 models predict suitable levels are yellow.

The distribution models with different sets of predictor variables [minimum, maximum and mean of either factorial aerobic scope (FAS), absolute aerobic scope (AAS) or temperature] predicted a similar core distribution for *C. laticeps* to the ϕ model (Figure S1.5 in the Supporting Information). The absolute aerobic scope (AAS) models however were unable to adequately delimit the warm edge of *C. laticeps’* distribution with more than 50% of random forest models predicting suitable habitat to occur in Mozambican tropical waters that does not support the robust occurrence data [ellipses, Figure S1.5 (d) in the Supporting Information]. Around the cooler, low oxygen western edge of *C. laticeps’* range, the factorial aerobic scope (FAS), absolute aerobic scope (AAS) and temperature only models predicted distributions to stretch past Cape Town into areas where catches of *C. laticeps* are not common (Fig. 6a–d). The ϕ distribution model most accurately delimited the spatial area where *C. laticeps* abundantly occurs across both edges of its known distribution, which was further supported by the ϕ distribution model iterations having a higher mean predictive accuracy over models with other predictor variables (Figure S1.6 in the Supporting Information). This difference in predictive accuracy among sets of distribution models was significant [one-way ANOVA, *F*(3,36) = 8.064, *P*-value < 0.0005] but only between AAS and ϕ or temperature predictor models (Tukey’s HSD, adjusted *P*-value < 0.05).

## Discussion

The geographic distribution of *C. laticeps* is constrained by a minimum critical metabolic index (ϕ_crit_) threshold of 2.78, whereby ϕ is limited in the west by supply (low oxygen) and in the east by demand (high temperatures). Importantly, we show that the distribution of *C. laticeps* is limited by different environmental variables across its contemporary distribution but through their influences on a common mechanism–aerobic metabolism.

**Figure 7:**
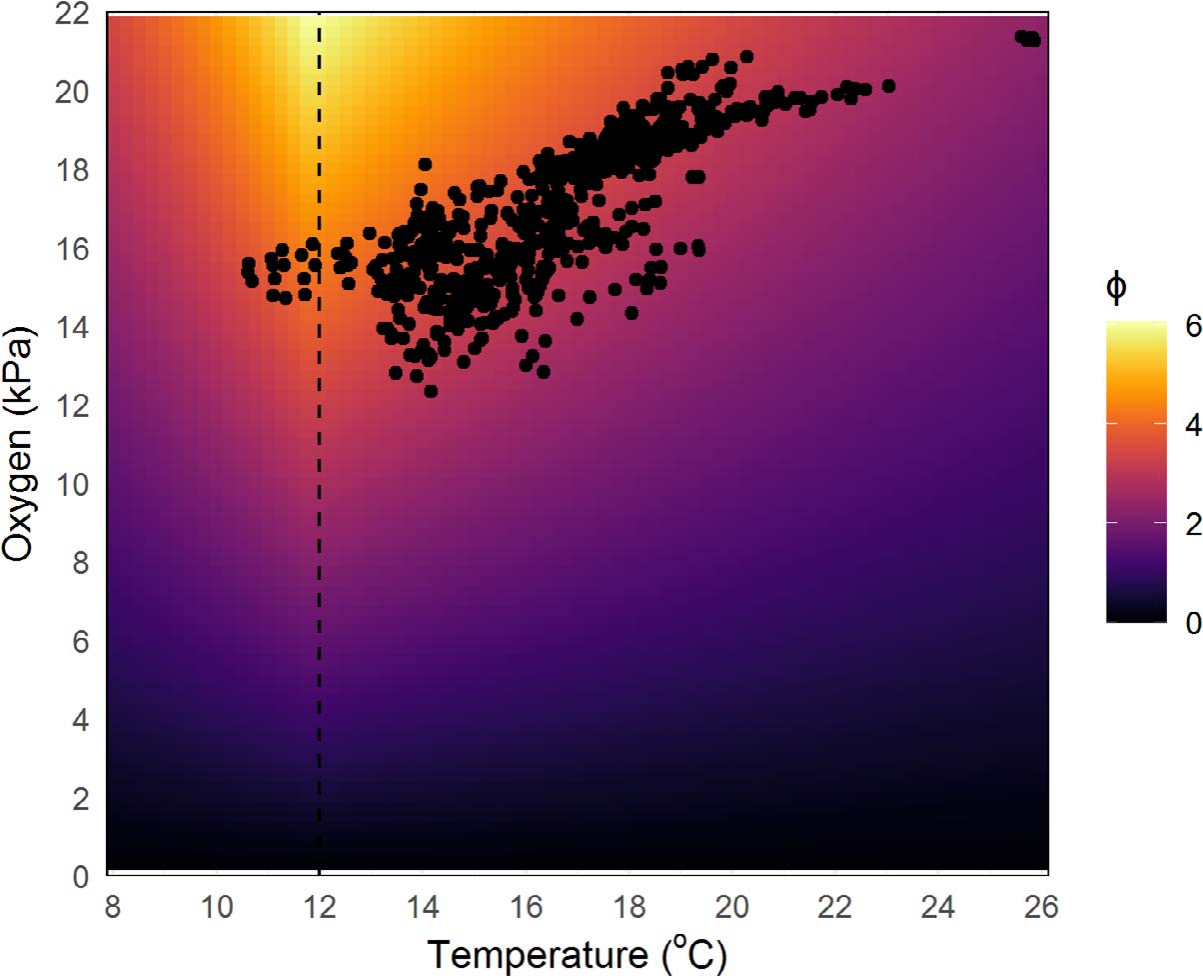
Temperature and oxygen availability associated with minimum metabolic index exposure for *Chrysoblephus laticeps*. Black points represent the temperature and oxygen availability associated with the minimum monthly metabolic index (ϕ) value per year (2005–2009), for grid cells where *C. laticeps* occurs. Colours represent ϕ values from the calibrated model for *C. laticeps*.

Identifying consistent patterns in physiological limits on species distributions across taxa is rare (Bozinovic *et al.*, 2011; Evans *et al.*, 2015). The monthly ϕ_crit_ value for *C. laticeps* (2.78) found here; however, is similar to the summer ϕ_crit_ (3.2) found for the related sharpsnout seabream (*Diplodus puntazzo*) in the north Atlantic (Deutsch *et al.*, 2015). Deutsch *et al*. (2015) do show consistent patterns between taxa regarding the warm equatorward limit of ϕ, with summer ϕ_crit_ ranging from 2.2 for common eelpout (*Zoarces viviparus*) to 3.3 for Atlantic cod (*Gadus morhua*), due to the antagonistic effect of rising temperature on ϕ through exponentially increasing oxygen demand. These results suggest that, on average, marine fishes require environmental conditions that permit a factorial aerobic scope of around three, although the number of fishes tested is small. Estimating *in situ* metabolic rates of wild fishes (field metabolic rates) remains a technological challenge (Treberg *et al.*, 2016). Recent advances in otolith isotope analysis have revealed that the average field metabolic rate (wild fishes) to standard metabolic rate (measured in the laboratory) ratio for Atlantic cod, *G. morhua*, ranged between 1.9 and 3.5 (Chung *et al.*, 2019). This isotope field metabolic rate work suggests that, on average, the wild metabolic rates of *G. morhua* will begin to be compromised if environmental conditions limit factorial aerobic scope below 3.5 which is comparable to annual ϕ_crit_ of 3.7 for *G. morhua* distribution (Deutsch *et al.*, 2015). The ϕ_crit_ will vary per species by lifestyle and morphology, but for low-energy teleost fishes that are not obligate swimmers, the aerobic supply to demand ratio of ~ 2–3.5 appears to be an important constraint (Deutsch *et al.*, 2015).

A bidirectional pattern between hypoxia tolerance and temperature has largely gone undetected, perhaps due to a disproportionate focus on warm-temperature experiments in an era of climate warming (Szekeres *et al.*, 2016). This pattern is new to fishes but has been reported for an intertidal Anthozoan cnidarian, *Diadumene lineata* (Boag *et al.*, 2018). A bidirectional relationship between hypoxia tolerance and temperature (e.g. Fig. 2a, 12°C) indicates that O_2crit_ is not primarily driven by temperature effects on increased oxygen demand (standard metabolic rate) but rather by the oxygen supply capacity and how it scales with temperature compared to the maximum metabolic rate (Seibel and Deutsch, 2020). The decrease in oxygen supply capacity at cold temperatures may be driven by the increased viscosity and thicker boundary layers of cold water that can impede oxygen diffusion rates (Verberk *et al.*, 2011). When such a bidirectional pattern is present, the utility of ϕ is extended beyond predicting only warm edge habitat limits and towards including both warm and cool edge limits across the entire distribution of an organism. In this case, however, there were few temperature points below 12°C associated with minimum annual ϕ for locations where *C. laticeps* occurs indicating that low oxygen associated with lower temperatures is primarily limiting ϕ (Fig. 7). When the temperature and oxygen availability associated with minimum ϕ layers where *C. laticeps* occurs are mapped, it mirrors the *p*O_2crit_–temperature bidirectional relationship. This correspondence highlights the interactive effects of both variables in setting the suitability of a habitat patch (Fig. 7). Given the increased utility of ϕ when a bidirectional O_2crit_–temperature relationship is present, we advocate for generating O_2crit_ data across the full range of temperatures experienced by an organism and at rates of exposure that are ecologically relevant, for future experimental physiology.

Biodiversity is affected by multiple dimensions of climate change across space and time that may not be captured in metrics of magnitude such as means (Garcia *et al.*, 2014; Waldock *et al.*, 2018). We find that the minimum monthly ϕ best explained the distribution limits of *C. laticeps* rather than means or maximums in all 10 random forest model runs. Whilst we rely on magnitudes (mean, maximum, minimum) of ϕ for this analysis, the monthly temporal resolution gives an indication of ϕ temporal availability (the total duration of a specific environmental event [e.g. Waldock *et al*., 2018)] that *C. laticeps* can tolerate. Whilst it is possible that monthly temporal resolution is too coarse to quantify absolute lower thresholds of ϕ, we did observe good recoveries of *C. laticeps* following acute (over 48 h) temperature and oxygen stressors in the lab, suggesting a capacity to withstand sub-optimal ϕ on a daily scale. The dimension of ‘time’ is an important consideration for future lab studies to calibrate ϕ as longer-term acclimation can reduce the temperature sensitivity of metabolic measurements (Semsar-kazerouni and Verberk, 2018; Bates and Morley, 2020). It is important that the rates of change in environmental stressors in the lab and the temporal resolution of spatial layers are ecologically relevant to accurately quantify organism responses to prevailing environmental change. At best, we can conclude that *C. laticeps* requires temperature and oxygen conditions that result in an average factorial aerobic scope of ~three, over monthly periods.

The effect of temperature on species distributions is well studied in the marine environment (e.g. Lasram *et al*., 2010; Albouy *et al*., 2013), but since oxygen is more difficult to measure in the field and manipulate in the laboratory, it has been neglected, despite its importance in limiting aerobic energy production in organisms (Bianucci *et al.*, 2016; Stortini *et al.*, 2017). For example, Verberk *et al.*, (2016) consider both variables and show that *in situ* oxygen availability influences the thermal suitability of a habitat patch for two Mayfly species, which corresponds to laboratory studies where temperature tolerance is reduced under hypoxia. The limited use of oxygen as a variable may be attributed to our inability to measure *in situ* oxygen levels from satellites and the lack of precision in oxygen data from the ocean models (Robinson *et al.*, 2011). Even so, recent advances in numerical ocean models have vastly enhanced information regarding ocean oxygen availability (Rose *et al.*, 2017), but improved predictions at finer temporal and spatial scales are still needed (Breitburg *et al.*, 2018). Even when accurate oxygen and temperature data are available, a complex non-linear interaction between the two variables (e.g. O_2crit_ ~ temperature_,_  Fig. 2a) on habitat suitability is often poorly represented by regression or envelope models (Golding and Purse, 2016). Advances in machine learning classification models can incorporate these non-linear complexities and overcome these difficulties (Elith *et al.*, 2006).

We advocate the use of physiological models to transform environmental data into indices of habitat suitability and then to combine these with correlative species distribution modelling (SDM) approaches to bridge the gap between correlation and process and filter out interaction complexities (Austin, 2007; Kearney *et al.*, 2008; Kearney and Porter, 2009; Dormann *et al.*, 2012; Rodríguez *et al.*, 2019). Physiological indicators, like aerobic scope, can project beyond the range of data and have been incorporated into predictive models for fisheries species (Peck *et al*. 2016; Marras *et al*. 2015). For example, Marras *et al*. (2015) used experimentally derived aerobic scopes of two fish species, the seabream (*Sarpa salpa*) and the marbled spinefoot (*Siganus rivulatus*), in the Mediterranean to develop a spatial thermal habitat suitability index and predict the distributions of both species. Cucco *et al*. (2012) developed an aerobic scope-based physiological model and combined it with temporally resolved oceanographic data to demonstrate that the migration of flathead mullet (*Mugil cephalus*) in the Mediterranean generally tracks the environmental conditions that optimize aerobic scope. Although these physiological-based mechanistic models overcome many of the limitations associated with purely correlative SDMs, they often assume that temperature is the only environmental variable that modulates how a fish population relates to its immediate environment (Sinclair *et al.*, 2016).

By including oxygen availability together with temperature, ϕ overcomes the temperature-dependence limitations of aerobic scope. Whilst the aforementioned aerobic scope models have predictive accuracy, an increasing number of studies have found mismatches between temperatures where aerobic scope is optimized versus other important ecological/physiological processes such as development, growth or thermal preference, highlighting that aerobic scope is not always a reliable indicator of potential climate responses (Clark *et al.*, 2013; Gräns *et al.*, 2014; Norin *et al.*, 2014). These aerobic scope–temperature mismatches usually occur at the warm edges of an organism’s thermal range (Jutfelt *et al.*, 2018). Our distribution modelling results support this notion as most of the absolute aerobic scope models over-predicted *C. laticeps*’ distribution into warm tropical waters, far from where it occurs naturally (Figure S1.5d in the Supporting Information). When aerobic scope models fail to explain ecological patterns, it may be because oxygen availability is not incorporated in a quantifiable way. Hypoxia has been shown to reduce the preferred temperature of teleost fish (Steffensen and Lomholt, 1991; Enders *et al.*, 2019), but whether this is mediated through hypoxia effects on thermal optima for aerobic scope remains to be investigated. It is worth noting that where aerobic scope is found not be ecologically relevant, the study species sometimes occur in hypoxic waters like the Atlantic halibut (*Hippoglossus hippoglossus*) (Gräns *et al.*, 2014) which occupies hypoxic areas in the north Atlantic Ocean (Breitburg *et al.*, 2018) or barramundi (*Lates calcarifer*) (Norin *et al.*, 2014), which are found in hypoxic mangroves in Australia, for example. However, this pattern is far from ubiquitous, and several studies based on normoxic species, such as the pink salmon (*Oncorhynchus gorbuscha*) (Clark *et al.*, 2011) or coho salmon (*Oncorhynchus kisutch*) (Raby *et al.*, 2016), have found absolute aerobic scope increases throughout temperatures that are not experienced by the species.

Distribution predictions for *C. laticeps* up to the end of the century indicate that the western and eastern edges will contract slightly due to the general warming trend along warm limits in the east and greater hypoxia in the west. Overall, the core distribution of *C. laticeps* is, however, predicted to persist up until at least 2100. This predicted contraction of *C. laticeps*’ distribution is consistent with climate-mediated distribution shifts of fish species already recorded off the temperate South African coastal zone (Whitfield *et al.*, 2016). The Cape anchovy (*Engraulis encrasicolus*) shifted its distribution from the southern Benguela to the Agulhas Bank during the mid-1990s, which was attributed to an increase in upwelling off the Agulhas Bank (Roy *et al*. 2007). Along the east coast, warming has resulted in a southward extension in distribution and abundance of tropical species (Mbande *et al.*, 1995; James *et al.*, 2008; Lloyd *et al.*, 2012; Whitfield *et al.*, 2016), which is consistent with the general poleward shift observed for fish species globally (Hastings *et al.*, 2020). The extension of cool-temperate habitat and species in the west and subtropical habitat and species in the east is predicted to put a ‘squeeze’ on potential distribution shifts of warm- and cool-temperate species around South Africa (James *et al.*, 2013; Potts *et al.*, 2015; Whitfield *et al.*, 2016) which is corroborated by the findings from this study. Whilst a predicted distribution contraction is a concern for any species, the most productive fishing grounds for *C. laticeps* in terms of catch per unit effort, west of Port Alfred and east of the Cape Town (Kerwath *et al.*, 2013) are predicted will persist up to 2100 according to our data.

In conclusion, we highlight that the metabolic index (ϕ) has the potential to explain the whole distribution of marine fish, if calibrated across a wide temperature range. We also show that ϕ can predict the distribution of an endemic marine organism across a dynamic heterogenous marine environment. Importantly, whilst we highlight how different environmental variables (oxygen and temperature) limit different edges of the distribution for *C. laticeps,* this is through a common mechanism. Studies investigating the effects of climate change on marine organism distributions should therefore consider the combined, interacting effects of oxygen and temperature to be ecologically relevant (Townhill *et al.*, 2017), and physiological indices such as ϕ offer a framework to achieve this.

## Ethics

Ethics for this research was approved under the regulations of Rhodes University Animal Ethics (DIFS152025) and the Animal Use and Care Committee of the South African National Parks (004/16).

## Supplementary material

Supplementary material is available at *Conservation Physiology* online.

## Supplementary Material

Supporting_Information_Revision_v2_coaa090

## Data Availability

Occurrence data for *Chrysoblephus laticeps* is available from the National Marine Linefish System at The Department of Environment, Forestry and Fisheries in South Africa. Ocean model data is housed at the National Oceanographic Centre, Southampton. Experimental data is available in the supplementary material and analysis scripts are available from the lead author upon request.

## References

[ref1] Albouy C, Leprieur F, Lasram FBR, Somot S, Aznar R, Velez L (2013) Projected climate change and the changing biogeography of coastal Mediterranean fishes. J Biogeogr 40: 534–547.

[ref2] Austin M (2007) Species distribution models and ecological theory: a critical assessment and some possible new approaches. Ecol Modell 200: 1–19.

[ref3] Barbet-Massin M, Jiguet F, Albert CH, Thuiller W (2012) Selecting pseudo-absences for species distribution models: how, where and how many? Methods Ecol Evol 3: 327–338.

[ref4] Bates AE et al. (2018) Biologists ignore ocean weather at their peril. Nature 560: 299–301. 30108353 10.1038/d41586-018-05869-5

[ref5] Bates AE, Morley SA (2020) Interpreting empirical estimates of experimentally derived physiological and biological thermal limits in ectotherms. Can J Zool 7–11.

[ref6] Bianucci L, Fennel K, Chabot D, Shackell N, Lavoie D (2016) Ocean biogeochemical models as management tools: a case study for Atlantic wolffish and declining oxygen. ICES J Mar Sci 73: 263–274.

[ref7] Birk MA (2016) presens: Interface for PreSens Fiber Optic Data In R Packag.

[ref8] Boag TH, Stockey RG, Elder LE, Hull PM, Sperling EA (2018) Oxygen, temperature and the deep-marine stenothermal cradle of Ediacaran evolution. Proc R Soc B Biol Sci 285. doi: 10.1098/rspb.2018.1724. PMC6304043 30963899

[ref9] Bozinovic F, Calosi P, Spicer JI (2011) Physiological correlates of geographic range in animals. Annu Rev Ecol Evol Syst 42: 155–179.

[ref10] Bozinovic F, Pörtner HO (2015) Physiological ecology meets climate change. Ecol Evol 5: 1025–1030. 25798220 10.1002/ece3.1403 PMC4364817

[ref11] Brander KM (2007) Global fish production and climate change. Proc Natl Acad Sci 104: 19709–19714. 18077405 10.1073/pnas.0702059104 PMC2148362

[ref12] Breiman L (2001) Random forests. Mach Learn 45: 5–32.

[ref13] Breitburg D et al. (2018) Declining oxygen in the global ocean and coastal waters. Science (80-) 359. doi: 10.1126/science.aam7240. 29301986

[ref14] Buxton CD (1987) Life History Changes of Two Reef Fish Species in Exploited and Unexploited Marine Environment in South Africa PhD thesis. Rhodes University.

[ref15] Buxton CD (1990) The reproductive biology of Chrysoblephus laticeps and C. cristiceps (Teleostei: Sparidae). J Zool 220: 497–511.

[ref16] Chabot D, Steffensen JF, Farrell AP (2016) The determination of standard metabolic rate in fishes. J Fish Biol 88: 81–121. 26768973 10.1111/jfb.12845

[ref17] Cheung WWL, Lam VWY, Sarmiento JL, Kearney K, Watson R, Pauly D (2009) Projecting global marine biodiversity impacts under climate change scenarios. Fish Fish 10: 235–251.

[ref18] Chung MT, Trueman CN, Godiksen JA, Holmstrup ME, Grønkjær P (2019) Field metabolic rates of teleost fishes are recorded in otolith carbonate. Commun Biol 2: 1–10. 30675522 10.1038/s42003-018-0266-5 PMC6338665

[ref19] Claireaux G, Chabot D (2016) Responses by fishes to environmental hypoxia: integration through Fry’s concept of aerobic metabolic scope. J Fish Biol 88: 232–251. 26768976 10.1111/jfb.12833

[ref20] Clark TD, Jeffries KM, Hinch SG, Farrell AP (2011) Exceptional aerobic scope and cardiovascular performance of pink salmon (Oncorhynchus gorbuscha) may underlie resilience in a warming climate. J Exp Biol 214: 3074–3081. 21865520 10.1242/jeb.060517

[ref21] Clark TD, Sandblom E, Jutfelt F (2013) Aerobic scope measurements of fishes in an era of climate change: respirometry, relevance and recommendations. J Exp Biol 216: 2771–2782. 23842625 10.1242/jeb.084251

[ref22] Clarke A (2019) Energy flow in growth and production. Trends Ecol Evol 34: 502–509. 30832986 10.1016/j.tree.2019.02.003

[ref23] Clarke A, Fraser KPP (2004) Why does metabolism scale with temperature? Funct Ecol 18: 243–251.

[ref24] Cucco A, Sinerchia M, Lefrançois C, Magni P, Ghezzo M, Umgiesser G, Perilli A, Domenici P (2012) A metabolic scope based model of fish response to environmental changes. Ecol Modell 237–238: 132–141.

[ref25] Cutler DR, Edwards TC, Beard KH, Cutler A, Hess KT, Gibson J, Lawler JJ (2007) Random forests for classification in ecology. Ecology 88: 2783–2792. 18051647 10.1890/07-0539.1

[ref26] DAFF (2016) Status of the South African Marine Fishery Resources. DAFF, Cape Town

[ref27] Deutsch C, Ferrel A, Seibel B, Portner HO, Huey RB (2015) Climate change tightens a metabolic constraint on marine habitats. Science (80-) 348: 1132–1136. 10.1126/science.aaa1605 26045435

[ref28] Dormann CF, Schymanski SJ, Cabral J, Chuine I, Graham C, Hartig F, Kearney M, Singer A (2012) Correlation and process in species distribution models : bridging a dichotomy. J Biogeogr 39: 2119–2131.

[ref29] Dulvy NK, Rogers SI, Jennings S, Stelzenmüller V, Dye SR, Skjoldal HR (2008) Climate change and deepening of the North Sea fish assemblage: a biotic indicator of warming seas. J Appl Ecol 45: 1029–1039.

[ref30] Duncan MI, Bates AE, James NC, Potts WM (2019) Exploitation may influence the climate resilience of fish populations through removing high performance metabolic phenotypes. Sci Rep 9. doi: 10.1038/s41598-019-47395-y. PMC6685998 31391481

[ref31] Elith J et al. (2006) Novel methods improve prediction of species ’ distributions from occurrence data. Ecography (Cop) 29: 129–151.

[ref32] Enders EC, Wall AJ, Svendsen JC (2019) Hypoxia but not shy-bold phenotype mediates thermal preferences in a threatened freshwater fish, Notropis percobromus. J Therm Biol 84: 479–487. 31466789 10.1016/j.jtherbio.2019.08.001

[ref33] Ern R (2019) A mechanistic oxygen- and temperature- limited metabolic niche framework. Philos Trans R Soc B 374. doi: 10.1098/rstb.2018.0540. PMC6606458 31203757

[ref34] Evans TG, Diamond SE, Kelly MW (2015) Mechanistic species distribution modelling as a link between physiology and conservation. Conserv Physiol 3. doi: 10.1093/conphys/cov056. PMC4778482 27293739

[ref35] Farrell AP (2016) Pragmatic perspective on aerobic scope: peaking, plummeting, pejus and apportioning. J Fish Biol 88: 322–343. 26592201 10.1111/jfb.12789

[ref36] Fry FEJ (1971) The effect of environmental factors on the physiology of fish In: Hoar WS, Randall DJ, eds. Fish Physiology, Vol 6 Environmental Relations and Behavior Academic Press, New York, pp 1–98.

[ref37] Garcia RA, Cabeza M, Rahbek C, Araújo MB (2014) Multiple dimensions of climate change and their implications for biodiversity, Science (80-). 344. doi: 10.1126/science.1247579. 24786084

[ref38] Golding N, Purse BV (2016) Fast and flexible Bayesian species distribution modelling using Gaussian processes. Methods Ecol Evol 7: 598–608.

[ref39] Goschen WS, Schumann EH (2011) The Physical Oceanographic Processes of Algoa Bay, with Emphasis on the Western Coastal Region. South African Environmental Observation Network (SAEON), Internal Report, South Africa.

[ref40] Götz A, Kerwath SE, Attwood CG, Sauer WHH (2008) Effects of fishing on population structure and life history of roman Chrysoblephus laticeps (Sparidae). Mar Ecol Prog Ser 362: 245–259.

[ref41] Gräns A et al. (2014) Aerobic scope fails to explain the detrimental effects on growth resulting from warming and elevated CO2 in Atlantic halibut. J Expermental Biol 217: 711–717. 10.1242/jeb.096743 24574386

[ref42] Grantham BA, Chan F, Nielsen KJ, Fox DS, Barth JA, Huyer A, Lubchenco J, Menge BA (2004) Upwelling-driven nearshore hypoxia signals ecosystem and oceanographic changes in the northeast Pacific. Nature 429: 749–754. 15201908 10.1038/nature02605

[ref43] Gruber N (2011) Warming up, turning sour, losing breath: ocean biogeochemistry under global change. Philos Trans R Soc A 369: 1980–1996. 10.1098/rsta.2011.0003 21502171

[ref44] Hastings RA et al. (2020) Climate change drives Poleward increases and equatorward declines in marine species report climate change drives poleward increases and equatorward declines in marine species. Curr Biol 30: 1–6. 32220327 10.1016/j.cub.2020.02.043

[ref45] Hijmans RJ (2016) raster: Geographic Data Analysis and Modeling In R Package.

[ref46] Horodysky AZ, Cooke SJ, Brill RW (2015) Physiology in the service of fisheries science: why thinking mechanistically matters. Rev Fish Biol Fish 25: 425–447.

[ref47] IPCC (2014) Climate Change 2014: Synthesis Report. Contribution of Working Groups I In II and III to the Fifth Assessment Report of the Intergovernmental Panel on Climate Change, IPCC, Geneva, Switzerland.

[ref48] James NC, Van Niekerk L, Whitfield AK, Potts WM, Götz A, Paterson AW (2013) Effects of climate change on South African estuaries and associated fish species. 57: 233–248.

[ref49] James NC, Whitfield AK, Cowley PD (2008) Preliminary indications of climate-induced change in a warm-temperate South African estuarine fish community. J Fish Biol 72: 1855–1863.

[ref50] Jarre A, Hutchings L, Crichton M, Wieland K, Lamont T, Blamey LK, Illert C, Hill E, Berg MVANDEN (2015) Oxygen-depleted bottom waters along the west coast of South Africa, 1950–2011. Fish Oceanogr 24: 56–73.

[ref51] Jutfelt F et al. (2018) Oxygen- and capacity-limited thermal tolerance: blurring ecology and physiology. J Exp Biol 221. doi: 10.1242/jeb.169615. 29321291

[ref52] Kearney M, Phillips BL, Tracy CR, Christian KA, Betts G, Porter WP (2008) Modelling species distributions without using species distributions: the cane toad in Australia under current and future climates. Ecography (Cop) 31: 423–434.

[ref53] Kearney M, Porter W (2009) Mechanistic niche modelling: combining physiological and spatial data to predict species’ ranges. Ecol Lett 12: 334–350. 19292794 10.1111/j.1461-0248.2008.01277.x

[ref54] Kelly D, Richards C (2018) Oce: analysis of oceanographic data. R Packag version 10–11.

[ref55] Kerwath SE, Götz A, Attwood C, Cowley P, Sauer W (2007a) Movement pattern and home range of Roman Chrysoblephus laticeps. African J Mar Sci 29: 93–103.

[ref56] Kerwath SE, Götz A, Attwood CG, Sauer WHH, Wilke CG (2007b) Area utilisation and activity patterns of roman Chrysoblephus laticeps (Sparidae) in a small marine protected area. African J Mar Sci 29: 259–270.

[ref57] Kerwath SE, Winker H, Götz A, Attwood CG (2013) Marine protected area improves yield without disadvantaging fishers. Nat Commun 4. doi: 10.1038/ncomms3347. 23962973

[ref58] Lasram FBR, Guilhaumon F, Albouy C, Somot S, Thuiller W, Moullot D (2010) The Mediterranean Sea as a ‘ cul-de-sac ’ for endemic fishes facing climate change. Glob Chang Biol 16: 3233–3245.

[ref59] Liaw A, Weiner M (2002) Classificataion and regression by randomForest. R News 2: 18–22.

[ref60] Lima FP, Wethey DS (2012) Three decades of high-resolution coastal sea surface temperatures reveal more than warming. Nat Commun 3. doi: 10.1038/ncomms1713. 22426225

[ref61] Link JS, Nye JA, Hare JA (2011) Guidelines for incorporating fish distribution shifts into a fisheries management context. Fish Fish 12: 461–469.

[ref62] Lloyd P, Plaganyi EE, Weeks SJ, Magno-Canto M, Plaganyi G (2012) Ocean warming alters species abundance patterns and increases species diversity in an African sub-tropical reef-fish community. Fish Oceanogr 21: 78–94.

[ref63] Lutjeharms JRE, Cooper J, Roberts M (2000) Upwelling at the inshore edge of the Agulhas current. Cont Shelf Res 20: 737–761.

[ref64] Madec G (2008) NEMO Ocean Engine. Institut Pierre-Simon Laplace (IPSL), France.

[ref64a] Marras S, Cucco A, Antognarelli F, Azzurro E, Milazzo M, Bariche M, Butenschön M, Kay S, Bitetto M Di, Quattrocchi G et al. (2015) Predicting future thermal habitat suitability of competing native and invasive fish species?: from metabolic scope to oceanographic modelling. Conservation Physiology 3: 1–14. doi: 10.1093/conphys/cou059. PMC4778460 27293680

[ref65] Mbande S, Whitfield A, Cowley P (1995) The ichthyofaunal composition of the Mngazi and Mngazana estuaries: a comparative study. Smithiana Bull 4: 1–20.

[ref66] Mi C, Huettmann F, Guo Y, Han X, Wen L (2017) Why choose Random Forest to predict rare species distribution with few samples in large undersampled areas? Three Asian crane species models provide supporting evidence. Peer J . doi: 10.7717/peerj.2849. PMC5237372 28097060

[ref67] Nelson JA (2016) Oxygen consumption rate v. rate of energy utilization of fishes: a comparison and brief history of the two measurements. J Fish Biol 88: 10–25. 26768970 10.1111/jfb.12824

[ref68] Norin T, Malte H, Clark TD (2014) Aerobic scope does not predict the performance of a tropical eurythermal fish at elevated temperatures. J Exp Biol 217: 244–251. 24115064 10.1242/jeb.089755

[ref69] Parmesan C (2006) Ecological and evolutionary responses to recent climate change. Annu Rev Ecol Evol Syst 37: 637–671.

[ref70] Peck MA et al. (2016) Projecting changes in the distribution and productivity of living marine resources: a critical review of the suite of modelling approaches used in the large European project VECTORS. Estuar Coast Shelf Sci 201: 40–55.

[ref71] Pecl GT et al. (2017) Biodiversity redistribution under climate change: impacts on ecosystems and human well-being. Science (80-) 355. doi: 10.1126/science.aai9214. 28360268

[ref72] Penn JL, Deutsch C, Payne JL, Sperling EA (2018) Temperature-dependent hypoxia explains biogeography and severity of end-Permian marine mass extinction. Science (80-) 362. doi: 10.1126/science.aat1327. 30523082

[ref73] Perry AL, Low PJ, Ellis JR, Reynolds JD (2005) Climate change and distribution shifts in marine fishes. Science (80-) 308: 1912–1915. 10.1126/science.1111322 15890845

[ref74] Pinsky ML, Eikeset AM, Mccauley DJ, Payne JL, Sunday JM (2019) Greater vulnerability to warming of marine versus terrestrial ectotherms. Nature 569: 108–111. 31019302 10.1038/s41586-019-1132-4

[ref75] Pinsky ML, Selden RL, Kitchel ZJ (2020) Climate-driven shifts in marine species ranges: scaling from organisms to communities. Ann Rev Mar Sci 12: 1–27. 10.1146/annurev-marine-010419-010916 31505130

[ref76] Poloczanska ES et al. (2013) Global imprint of climate change on marine life. Nat Clim Chang 3: 919–925.

[ref77] Popova E, Yool A, Byfield V, Cochrane K, Coward AC, Salim SS, Gasalla MA, Henson SA, Hobday AJ, Pecl GT (2016) From global to regional and back again: common climate stressors of marine ecosystems relevant for adaptation across five ocean warming hotspots. Glob Chang Biol 22: 2038–2053. 26855008 10.1111/gcb.13247 PMC4999053

[ref78] Portner HO, Farrell AP (2008) Physiology and climate change. Science (80-) 322: 690–692. 10.1126/science.1163156 18974339

[ref79] Potts WM, Götz A, James NC (2015) Review of the projected impacts of climate change on coastal fishes in southern Africa. Rev Fish Biol Fish 25: 603–630.

[ref80] Pretorius M, Huggett JA, Gibbons MJ (2016) Summer and winter differences in zooplankton biomass, distribution and size composition in the KwaZulu-Natal Bight, South Africa. African J Mar Sci 38: Supplem: S155–S168.

[ref81] Raby GD, Casselman MT, Cooke SJ, Hinch SG, Farrell AP, Clark TD (2016) Aerobic scope increases throughout an ecologically relevant temperature range in coho salmon. J Exp Biol 219: 1922–1931. 27059065 10.1242/jeb.137166

[ref82] Richards JG (2009) Metabolic and molecular responses of fish to hypoxia In JG Richards, AP Farrell, CJ Brauner, eds, Fish Physiology. Elsevier, Amsterdam, pp. 443–485.

[ref83] Roberts MJ (2005) Chokka squid (Loligo vulgaris reynaudii) abundance linked to changes in South Africa’s Agulhas Bank ecosystem during spawning and the early life cycle. ICES J Mar Sci 62: 33–55.

[ref84] Robinson LM, Elith J, Hobday AJ, Pearson RG, Kendall BE, Possingham HP, Richardson AJ (2011) Pushing the limits in marine species distribution modelling : lessons from the land present challenges. Glob Ecol Biogeogr 20: 789–802.

[ref85] Rodríguez L, García JJ, Carreño F, Martínez B, José J, Francisco G, Brezo C (2019) Integration of physiological knowledge into hybrid species distribution modelling to improve forecast of distributional shifts of tropical corals. Divers Distrib 25: 715–728.

[ref86] Rose KA, Dubravko J, Katja F, Hetland RD (2017) Modeling coastal hypoxia: Numerical simulations of patterns, controls and effects of dissolved oxygen dynamics In D Justic, K Rose, R Hetland, K Fennel, eds, Modeliing Coastal Hypoxia. Springer, Cham, pp. 1–433.

[ref87] Rouault M, Penven P, Pohl B (2009) Warming in the Agulhas current system since the 1980’s. Geophys Res Lett 36: 2–6.

[ref88] Rouault M, Pohl B, Penven P (2010) Coastal oceanic climate change and variability from 1982 to 2009 around South Africa. African J Mar Sci 32: 237–246.

[ref89] Roy C, van der Lingen C, Coetzee J, Lutjeharms J (2007) Abrupt environmental shift associated with changes in the distribution of cape anchovy Engraulis encrasicolus spawners in the southern Benguela. African J Mar Sci 29: 309–319.

[ref90] Santos F, Gomez-gesteira M, Alvarez I (2012) Differences in coastal and oceanic SST trends due to the strengthening of coastal upwelling along the Benguela current system. Cont Shelf Res 34: 79–86.

[ref91] Seibel BA, Deutsch C (2020) Oxygen supply capacity in animals evolves to meet maximum demand at the current oxygen partial pressure regardless of size or temperature. J Exp Biol 223: jeb210492. 32376709 10.1242/jeb.210492

[ref92] Semsar-kazerouni M, WCEP V (2018) It’s about time: linkages between heat tolerance, thermal acclimation and metabolic rate at different temporal scales in the freshwater amphipod Gammarus fossarum Koch, 1836. J Therm Biol 75: 31–37. 30017049 10.1016/j.jtherbio.2018.04.016

[ref93] Sinclair BJ et al. (2016) Can we predict ectotherm responses to climate change using thermal performance curves and body temperatures? Ecol Lett 19: 1372–1385. 27667778 10.1111/ele.12686

[ref94] Somero GN, Beers JM, Chan F, Hill TM, Klinger T, Litvin SY (2016) What changes in the carbonate system, oxygen, and temperature portend for the Northeastern Pacific Ocean: a physiological perspective. Bioscience 66: 14–26.

[ref95] Steffensen JF, Lomholt JP (1991) The influence of hypoxia on the preferred temperature of rainbow trout Oncorhynchus Mykiss. J Exp Biol 157: 75–86.

[ref96] Stortini CH, Chabot D, Shackell NL (2017) Marine species in ambient low-oxygen regions subject to double jeopardy impacts of climate change. Glob Chang Biol 23: 2284–2296. 27753179 10.1111/gcb.13534

[ref97] Sunday JM, Bates AE, Dulvy NK (2012) Thermal tolerance and the global redistribution of animals. Nat Clim Chang 2: 686–690.

[ref98] Szekeres P, Eliason EJ, Lapointe D, Donaldson MR, Brownscombe JW, Cooke SJ (2016) On the neglected cold side of climate change and what it means to fish. Clim Res 69: 239–245.

[ref100] Townhill BL, Van Der Molen J, Metcalfe JD, Simpson SD, Farcas A, Pinnegar JK (2017) Consequences of climate-induced low oxygen conditions for commercially important fish. Mar Ecol Prog Ser 580: 191–204.

[ref101] Treberg JR, Killen SS, MacCormack TJ, Lamarre SG, Enders EC (2016) Estimates of metabolic rate and major constituents of metabolic demand in fishes under field conditions: methods, proxies, and new perspectives. Comp Biochem Physiol -Part A Mol Integr Physiol 202: 10–22. 10.1016/j.cbpa.2016.04.022 27139083

[ref102] Ultsch GR, Regan MD (2019) The utility and determination of Pcrit in fishes. J Exp Biol 222. doi: 10.1242/jeb.203646. 31722971

[ref103] Verberk WCEP, Bilton DT, Calosi P, Spicer JI (2011) Oxygen supply in aquatic ectotherms: partial pressure and solubility together explain biodiversity and size patterns. Ecology 92: 1565–1572. 21905423 10.1890/10-2369.1

[ref104] Verberk WCEP, Overgaard J, Ern R, Bayley M, Wang T, Boardman L, Terblanche JS (2016) Does oxygen limit thermal tolerance in arthropods? A critical review of current evidence. Comp Biochem Physiol - Part A 192: 64–78. 10.1016/j.cbpa.2015.10.020 PMC4717866 26506130

[ref105] Waldock C, Dornelas M, Bates AE (2018) Temperature-driven biodiversity change: disentangling space and time. Bioscience 68: 873–884. 30464352 10.1093/biosci/biy096 PMC6238962

[ref106] Walther G, Post E, Convey P, Menzel A, Parmesan C, Beebee TJC, Fromentin J, OH I, Bairlein F (2002) Ecological responses to recent climate change. Nature 416: 389–395. 11919621 10.1038/416389a

[ref107] Welling SH, Refsgaard HHF, Brockhoff PB, Line H (2016) Forest floor visualizations of random forests. arXiv 160509196.

[ref108] Whitfield AK, James NC, Lamberth SJ, Adams JB, Perissinotto R, Rajkaran A, Bornman TG (2016) The role of pioneers as indicators of biogeographic range expansion caused by global change in southern African coastal waters. Estuar Coast Shelf Sci 172: 138–153.

[ref109] Yool A, Popova EE, Anderson TR (2013) M EDUSA-2.0: an intermediate complexity biogeochemical model of the marine carbon cycle for climate change and ocean acidification studies. Geosci Model Dev 6: 1767–1811.

[ref110] Yool A, Popova EE, Coward AC (2015) Future change in ocean productivity: is the Arctic the new Atlantic. J Geophys Res Ocean 120: 7771–7790.

