# Supplementary material for: Different drivers, common mechanism; the distribution of a reef fish is restricted by local-scale oxygen and temperature constraints on aerobic metabolism
Source: Conserv Physiol. 2020 Oct 26;8(1):coaa090. doi: 10.1093/conphys/coaa090 (PMC7904075; doi:10.1093/conphys/coaa090)
Supplement: Supporting_Information_Revision_v2_coaa090 [file supporting_information_revision_v2_coaa090.docx]

**Supporting Information**

**
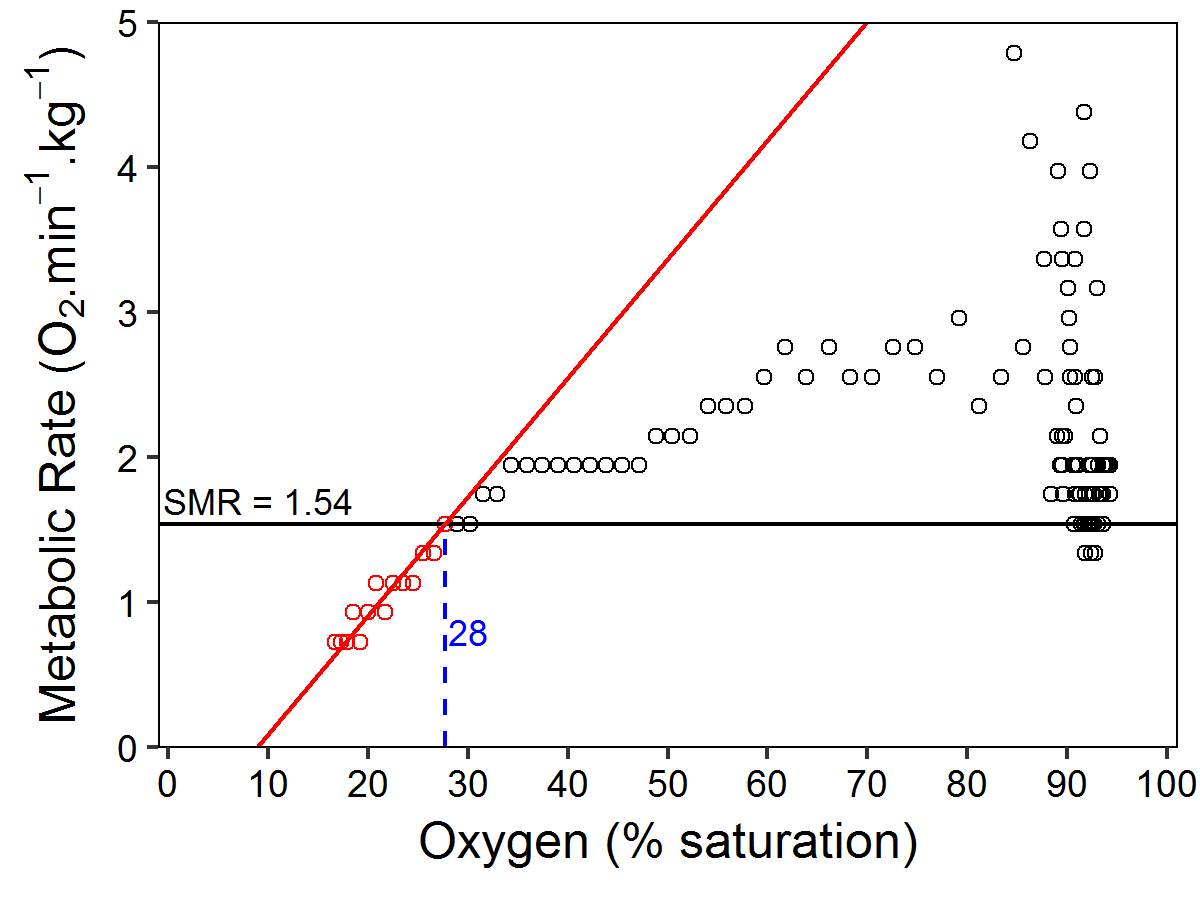
**

**Figure S1.1.** Example of how O_2crit_ was determined for an individual specimen at a set temperature. Each circle represents a metabolic rate at an O_2_ saturation. The solid black line is SMR calculated as the 0.2 percentile of metabolic rates in normoxia (>70%) during the intermittent flow respirometry cycle. The solid red line is the linear relationship between metabolic rate and O_2_ saturation for metabolic rates below O_2crit_. The intersection of both solid lines is the critical oxygen saturation (dotted blue line).

**Table S1.1.** Individual data from laboratory experiments to estimate *p*O_2Crit_ across temperatures. For each individual we recorded a unique identification (id), the test temperature (temp, °C), the critical oxygen saturation (O2crit, % saturation), the number of points included in the regression below smr (reg_pts), mass (kg), sampling location (site, PE = Port Elizabeth, TNP = Tsitsikamma National Park), sex (M = male, F = female, F/I = female/intersex, M/I = male/intersex), fork length (fl, cm) and total length (tl, cm) together with standard metabolic rate (smr, mgO_2_.min^-1^.kg^-1^) and maximum metabolic rate (mmr, mgO_2_.kg^-1^) both mass standardised with scaling exponents of 0.97 and 0.82 respectively from Duncan et al. (2019).

| **id** | **temp** | **O2crit** | **reg_pts** | **mass** | **site** | **sex** | **fl.cm** | **tl.cm** | **gsi** | **smr** | **mmr** |
| --- | --- | --- | --- | --- | --- | --- | --- | --- | --- | --- | --- |
| R1 | 16 | 28 | 15 | 1.3239 | PE | M | 34.5 | 36 | 0.2125 | 1.842805 | 5.15796 |
| R10 | 20 | 38.9 | 15 | 0.85833 | PE | M | 31.7 | 34.2 | 0.0814 | 3.644124 | 6.205527 |
| R11 | 20 | 26.2 | 15 | 1.55513 | TNP | M | 37.5 | 41.5 | 0.2092 | 1.881915 | 5.193935 |
| R12 | 20 | 42.3 | 15 | 0.83 | TNP | F/I | 32 | 34.5 | 0.6731 | 3.596209 | 6.384345 |
| R17 | 24 | 31.9 | 15 | 0.78287 | PE | F | 30.5 | 33 | 0.4829 | 2.304662 | 6.047625 |
| R18 | 24 | 40.5 | 15 | 0.4028 | PE | F | 24.5 | 27 | 0.5098 | 4.145451 | 6.288763 |
| R19 | 24 | 31.5 | 15 | 0.3209 | TNP | NA | NA | NA | NA | 2.462443 | 6.69932 |
| R20 | 24 | 45.3 | 15 | 0.9766 | TNP | F/I | 32.7 | 34.2 | 0.5325 | 3.567573 | 5.743843 |
| R21 | 16 | 23 | 15 | 0.48012 | TNP | F/I | 27 | 29 | 0.7595 | 1.447017 | 5.933034 |
| R22 | 16 | 20.5 | 15 | 1.171 | TNP | M | 34.7 | 37.2 | 0.1899 | 1.035713 | 4.967335 |
| R23 | 16 | 18.9 | 15 | 1.406 | PE | M | 37 | 40 | 0.1053 | 1.113239 | 4.643564 |
| R24 | 16 | 13.1 | 15 | 0.54 | PE | F/I | 27.2 | 29 | 0.7642 | 0.957737 | 5.07811 |
| R25 | 20 | 17.5 | 15 | 0.526 | PE | F/I | 26.5 | 28.5 | 0.4435 | 1.58944 | 4.813844 |
| R26 | 20 | 30.1 | 15 | 1.016 | PE | M | 33.2 | 35.6 | 0.1644 | 2.038829 | 5.748209 |
| R28 | 20 | 26.4 | 4 | 0.86217 | TNP | F/I | 32 | 34 | 0.7392 | 1.989394 | 6.202972 |
| R29 | 12 | 13.3 | 15 | 0.473 | TNP | F/I | 26.6 | 28.5 | 0.4334 | 0.676882 | 4.295113 |
| R3 | 16 | 28.8 | 15 | 1.4675 | TNP | M | 36 | 39.5 | 0.1803 | 1.295304 | 4.589474 |
| R30 | 12 | 13.2 | 15 | 0.899 | TNP | F/I | 32 | 34.8 | 0.4572 | 0.555909 | 4.852856 |
| R31 | 12 | 19.2 | 15 | 0.69676 | PE | M/I | 29.8 | 31.5 | 0.1059 | 1.259431 | 3.861003 |
| R32 | 12 | 16.5 | 15 | 0.435 | PE | F | 25 | 26.9 | 0.5649 | 1.185481 | 3.920831 |
| R33 | 24 | 34.6 | 4 | 0.652 | PE | F | 29 | 31.2 | 0.4232 | 2.573217 | 4.718723 |
| R34 | 24 | 44.2 | 4 | 1.014 | PE | M/I | 33.2 | 35.5 | 0.097 | 3.435186 | 6.269687 |
| R35 | 24 | 37 | 15 | 0.50764 | TNP | F/I | 26.5 | 28.5 | 0.7117 | 2.631722 | 7.116944 |
| R36 | 24 | 30.7 | 15 | 0.8367 | TNP | F/I | 31.2 | 34.1 | 0.5592 | 2.028069 | 6.549064 |
| R37 | 8 | 33.2 | 15 | 1.503 | TNP | M | 35 | 38.4 | 0.1461 | 1.026893 | 2.100885 |
| R39 | 8 | 38.2 | 15 | 1.243 | PE | M/I | 34.7 | 37.7 | 0.1387 | 0.958846 | 2.623498 |
| R4 | 16 | 21 | 15 | 0.7605 | TNP | F | 30.5 | 31.5 | 0.4313 | 1.479873 | 5.79272 |
| R40 | 8 | 20.6 | 15 | 0.618 | PE | F/I | 28.5 | 31.4 | 0.1339 | 0.776112 | 2.06274 |
| R42 | 20 | 25.1 | 15 | 0.644 | PE | F | 28.4 | 30.4 | 0.8213 | 1.829181 | 4.820881 |
| R43 | 20 | 28.1 | 15 | 0.86942 | TNP | F/I | 33.1 | 35.6 | 0.5769 | 1.561278 | 5.152352 |
| R44 | 20 | 35.2 | 15 | 0.5627 | TNP | F/I | 27.5 | 30.2 | 0.7724 | 2.059205 | 5.949325 |
| R46 | 16 | 28 | 15 | 0.884 | TNP | F | 31 | 33.5 | 0.8088 | 1.535455 | 4.484623 |
| R47 | 16 | 32.5 | 15 | 1.169 | PE | M | 33.5 | 36.5 | 0.0909 | 1.632637 | 4.019516 |
| R48 | 16 | 22.6 | 15 | 0.487 | PE | F | 26 | 28 | 0.8165 | 1.681248 | 3.470188 |
| R50 | 24 | 36.8 | 4 | 1.5 | PE | NA | 37 | 39 | NA | 2.738014 | 4.868969 |
| R52 | 24 | 30.6 | 4 | 0.9524 | TNP | F/I | 31.5 | 34.2 | 0.6144 | 2.512642 | 6.407642 |
| R6 | 12 | 21 | 15 | 1.0141 | TNP | F/I | 33 | 34.7 | 0.7363 | 1.003108 | 4.715572 |
| R7 | 12 | 17.4 | 15 | 1.1335 | PE | M | 35 | 37 | 0.0536 | 1.054034 | NA |
| R8 | 12 | 11.1 | 15 | 0.762 | PE | F/I | 28 | 30.5 | 0.3642 | 0.866686 | 4.207471 |

There was a weak positive relationship between mass and *p*O2crit, with the mass scaling exponent estimated at 0.17 (Figure S1.2). The size range tested here was small and extrapolation of results beyond this size range is cautioned.


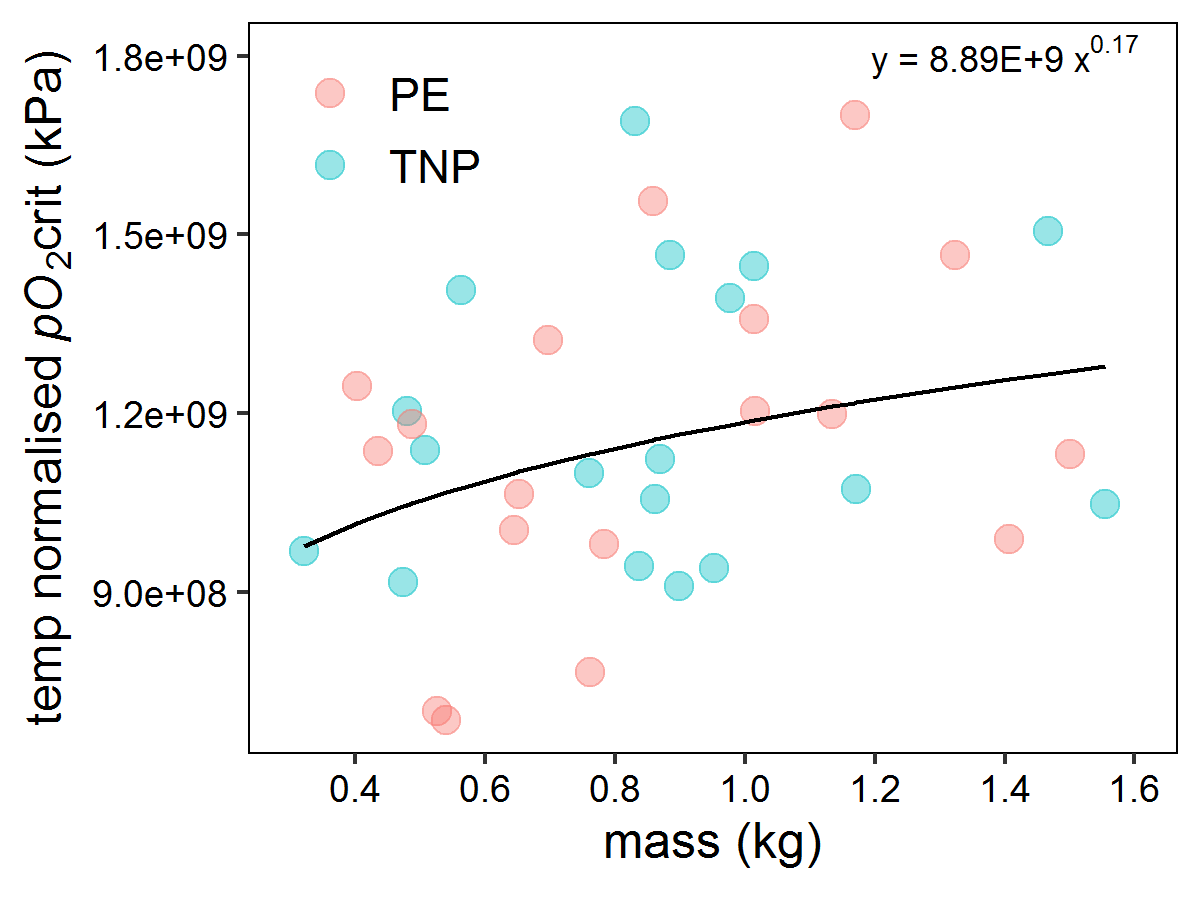


**Figure S1.2.** Estimating mass scaling exponent. Power law relationship (black line) between mass (kg) and temperature normalised *p*O_2_crit (Torr), indicating a mass scaling exponent of 0.17 for combined Tsitsikamma National Park (TNP) and Port Elizabeth (PE) sampling areas.

There was a positive linear relationship between *p*O2crit and temperature (12–24 °C), which was not significantly different between sampling populations (Table S1.2 *p*-value > 0.05, Figure S1.2). Although there was a negative relationship between *p*O2crit and temperature between 8–12 °C, the small sample size (n = 3) at 8 °C prohibited further analysis of differences between sampling populations for this temperature range. Consequently, all data were pooled to calibrate ϕ for *C. laticeps*.


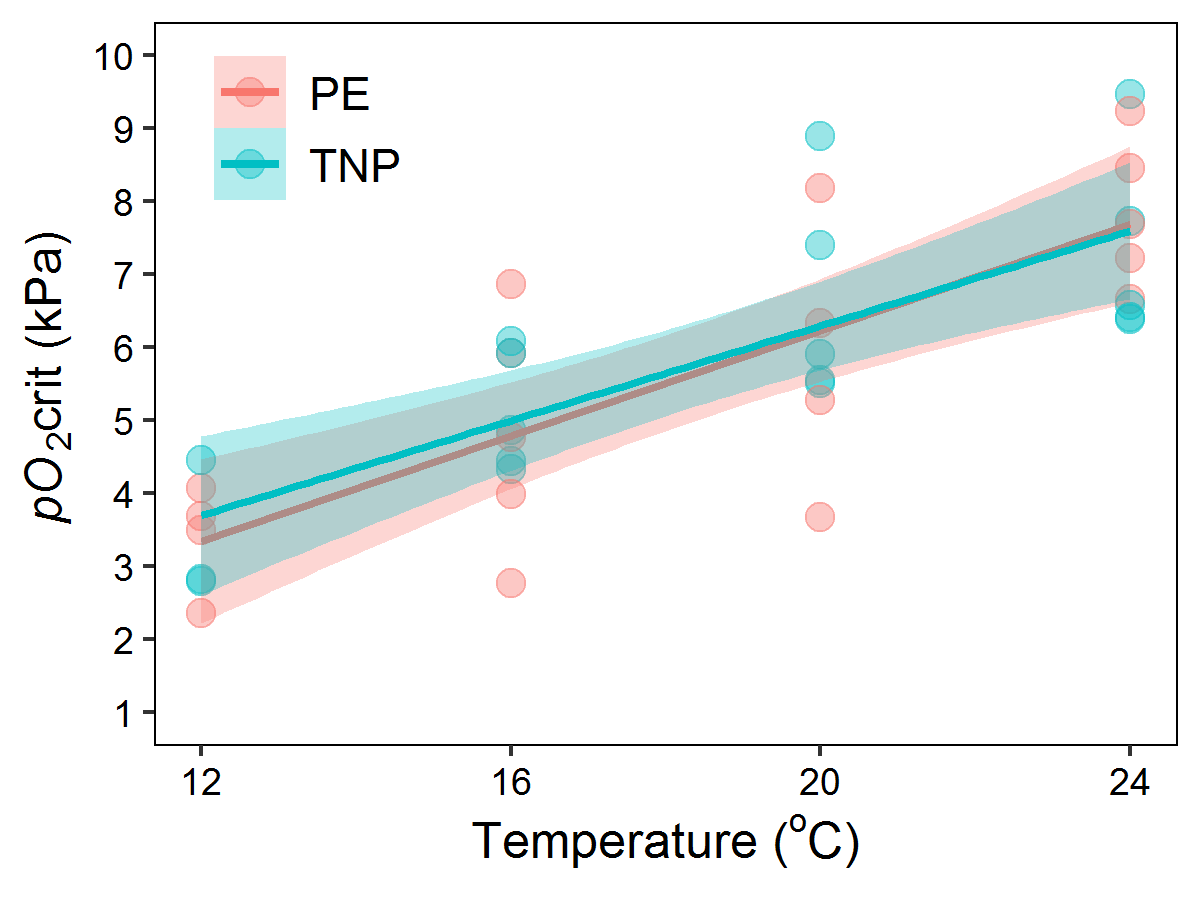


**Figure S1.3.** No sampling site differences in *p*O2crit. Temperature effect on mass normalised critical oxygen partial pressure (*p*O2crit) per Tsitsikamma National Park (TNP) and Port Elizabeth (PE) sampling area. Points represent individual data points fit with the linear relationship (solid line) and 95% confidence interval (shaded) for each sampling population.

**Table S1.2.** Linear model results for *p*O2crit as a function of temperature, sampling location (site) and their interaction. Significant *p*-values are highlighted in bold.

| Effect | Estimate | Std. Error | | t-value | | p-value | |
| --- | --- | --- | --- | --- | --- | --- | --- |
| intercept | -0.99 | 1.23 | | -0.81 | | 0.42 | |
| temperature | 0.36 | 0.07 | | 5.53 | | **< 0.00** | |
| site | 0.79 | 1.81 | | 0.44 | | 0.66 | |
| temperature: site | -0.04 | 0.10 | | -0.39 | | 0.70 | |
| R^2^ | 0.62 |  | |  | |  | |
| residual std. Error | 1.24 |  |  | |  | |  |

There was a positive linear relationship between *p*O2crit and temperature (12–24 °C), which was also not significantly different between different sex stages (Table S1.3 *p*-value > 0.05, Figure S1.3). Consequently, all data were pooled to calibrate ϕ for *C. laticeps*.


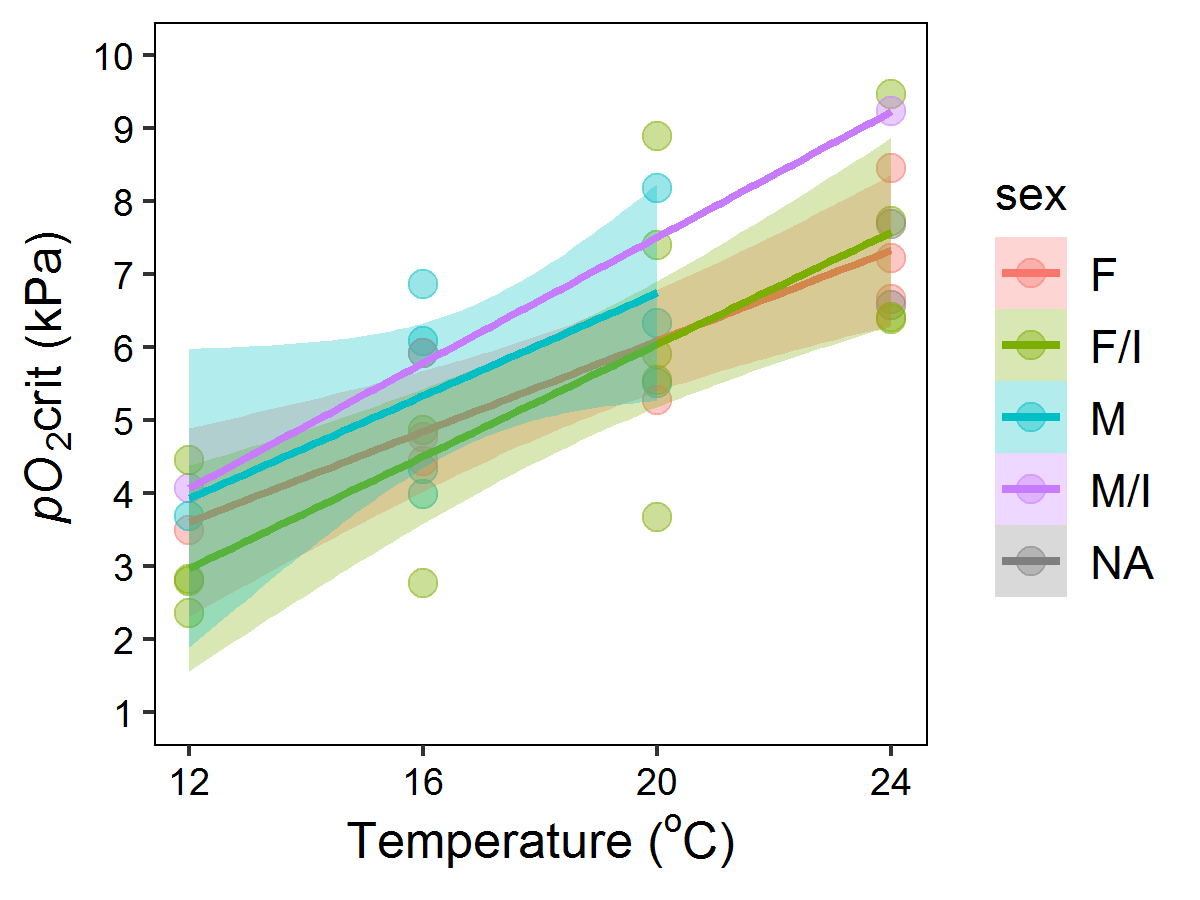


**Figure S1.4.** No sex-stage differences in *p*O2crit. Temperature effect on critical oxygen partial pressure (*p*O_2crit_) per sexual stage of test specimen (F = female, M = male, M/I = possibly intersex but male dominant, F/I = possibly intersex but female dominant. Points represent individual data points fit with the linear relationship (solid line) and 95% confidence interval (shaded) for each visually identified sexual stage.

**Table S1.3.** Linear model results for *p*O2crit as a function of temperature, sexual stage (sex) and their interaction. Significant *p*-values are highlighted in bold.

| Effect | Estimate | Std. Error | | t-value | | p-value | |
| --- | --- | --- | --- | --- | --- | --- | --- |
| intercept | -0.11 | 2.00 | | -0.06 | | 0.96 | |
| temperature | 0.31 | 0.10 | | 3.03 | | **0.01** | |
| sex (F/I) | -1.52 | 2.41 | | -0.63 | | 0.53 | |
| sex (M) | -0.18 | 3.49 | | -0.05 | | 0.96 | |
| sex (M/I) | -0.98 | 3.46 | | -0.28 | | 0.78 | |
| temperature: sex (F/I) | 0.07 | 0.12 | | 0.59 | | 0.56 | |
| temperature: sex (M) | 0.04 | 0.20 | | 0.22 | | 0.83 | |
| temperature: sex (M/I) | 0.12 | 0.18 | | 0.66 | | 0.51 | |
| R2 | 0.67 |  |  | |  | |  |
| residual std. Error | 1.26 |  |  | |  | |  |

**Table S1.4.** Random Forest model performance for each run on a unique combination of data. Models rf1 to rf10 were run with a full set of predictor variables (mean, maximum and minimum ϕ and depth) with top two predictor performance, based on the mean decrease in accuracy, highlighted bold. Models rf11 to rf20 were run with only minimum ϕ and depth as predictors. All models’ predictive accuracy was assessed by sensitivity, specificity and overall accuracy of classifying the test data set.

| Model | Mean Decrease in Accuracy | | | |  | | Model Predictive Performance | | | |  |
| --- | --- | --- | --- | --- | --- | --- | --- | --- | --- | --- | --- |
|  | Mean ɸ | Maximum ɸ | Minimum ɸ | Depth | |  | | Sensitivity | Specificity | Accuracy | |
| rf1 | 33.45 | 27.62 | 53.23 | 60.57 | |  |  | 0.93 | 0.88 | 0.90 | |
| rf2 | 35.84 | 21.40 | 51.88 | 58.26 | |  | | 0.88 | 0.88 | 0.88 | |
| rf3 | 29.61 | 30.98 | 52.68 | 79.37 | |  | | 0.93 | 0.85 | 0.89 | |
| rf4 | 37.01 | 28.73 | 48.57 | 85.12 | |  | | 0.85 | 0.85 | 0.85 | |
| rf5 | 33.74 | 26.84 | 53.85 | 64.25 | |  | | 0.90 | 0.92 | 0.91 | |
| rf6 | 39.26 | 31.09 | 48.50 | 74.33 | |  | | 0.88 | 0.88 | 0.88 | |
| rf7 | 36.38 | 29.44 | 43.55 | 66.82 | |  | | 0.90 | 0.85 | 0.88 | |
| rf8 | 35.18 | 29.32 | 47.48 | 69.72 | |  | | 0.90 | 0.85 | 0.88 | |
| rf9 | 33.15 | 28.46 | 51.43 | 71.15 | |  | | 0.90 | 0.90 | 0.90 | |
| rf10 | 36.02 | 25.69 | 41.77 | 82.08 | |  | | 0.90 | 0.83 | 0.87 | |
|  |  |  |  |  | |  |  |  |  |  | |
| rf11 |  |  | 97.43 | 74.81 | |  | | 0.93 | 0.88 | 0.90 | |
| rf12 |  |  | 99.01 | 66.39 | |  | | 0.88 | 0.95 | 0.91 | |
| rf13 |  |  | 92.92 | 73.87 | |  | | 0.90 | 0.85 | 0.88 | |
| rf14 |  |  | 97.57 | 75.64 | |  | | 0.98 | 0.88 | 0.93 | |
| rf15 |  |  | 94.41 | 71.36 | |  | | 0.93 | 0.93 | 0.93 | |
| rf16 |  |  | 90.24 | 75.00 | |  | | 0.93 | 0.87 | 0.90 | |
| rf17 |  |  | 96.12 | 82.44 | |  | | 0.90 | 0.85 | 0.88 | |
| rf18 |  |  | 95.85 | 67.08 | |  | | 0.88 | 0.98 | 0.93 | |
| rf19 |  |  | 95.83 | 71.36 | |  | | 0.80 | 0.95 | 0.88 | |
| rf20 |  |  | 89.89 | 69.10 | |  | | 0.90 | 0.88 | 0.89 | |


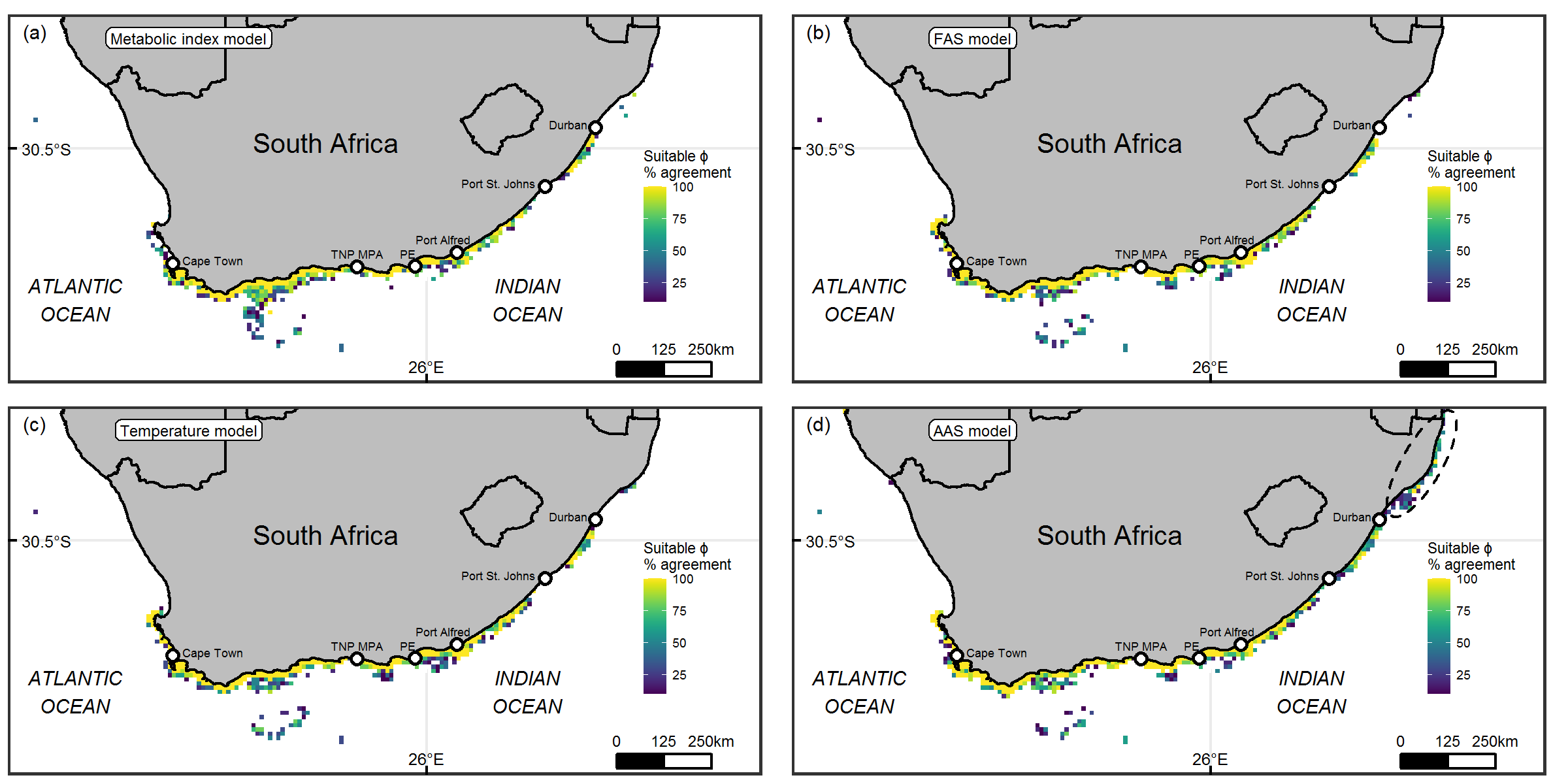
**Figure S1.5.** Absolute aerobic scope (AAS) (d) distribution model overpredicts the eastern edge of *Chrysoblephus laticeps* distribution into tropical waters where it doesn’t occur (dashed ellipses) compared to metabolic index (a), factorial aerobic scope (FAS) (b) and temperature (c) models.


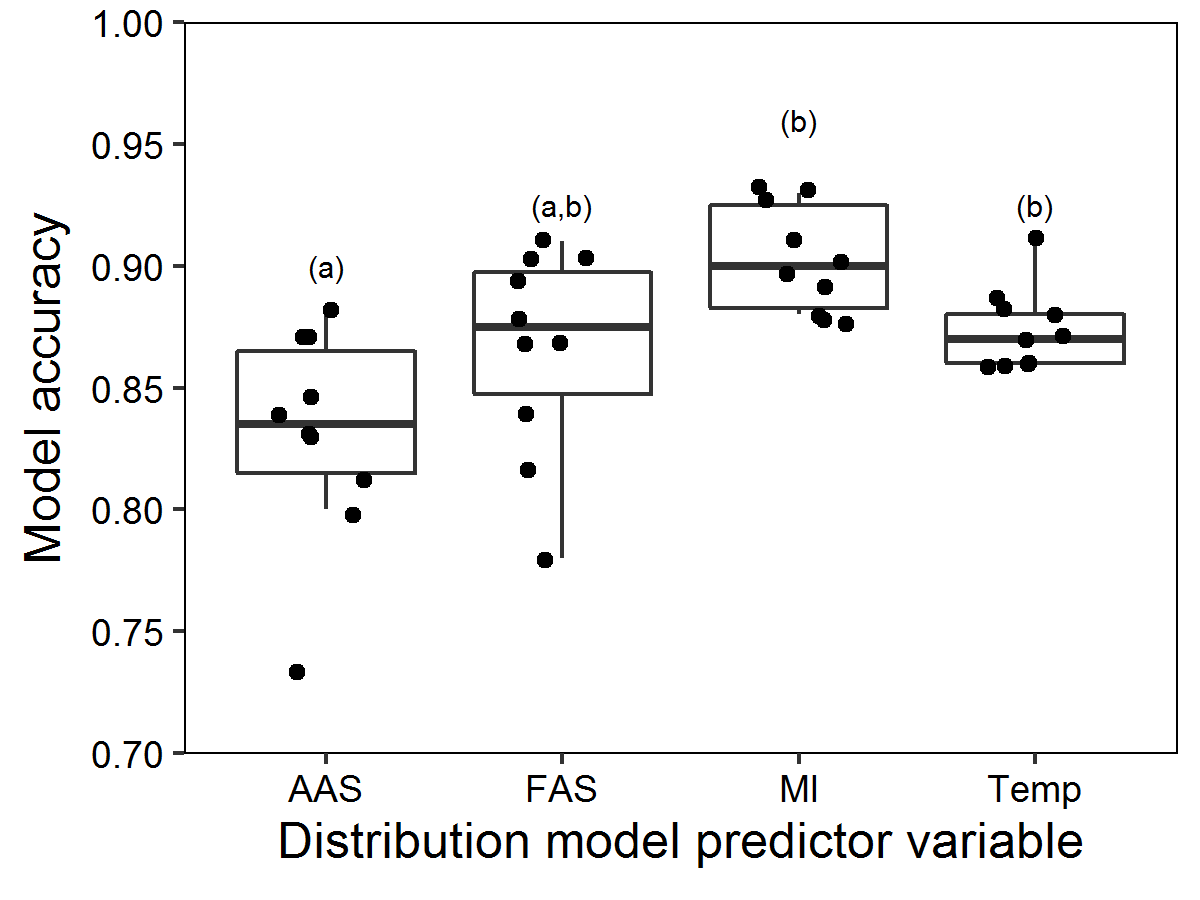


**Figure S1.6.** Random forest distribution model accuracy for each of the ten model runs (black dots) with either absolute aerobic scope (AAS), factorial aerobic scope (FAS), metabolic index (MI) or temperature (Temp) as predictor variables. Different letters indicate significant differences between the means tested with analysis of variance and Tukey’s post hoc test
